# Diagnostic approach to episodic ataxia types 1 and 2: a proposed algorithm for limited resource-settings

**DOI:** 10.3389/fneur.2026.1735246

**Published:** 2026-04-21

**Authors:** Claudio M. de Gusmao, Lucas H. M. R. Garcia, Jonathan W. Mink, Alex R. Paciorkowski, Tamara Pringsheim, Bruno Assis Della Ripa, Carolina Silva Rauffus, Ana Carolina Coan, Laura Silveira-Moriyama

**Affiliations:** 1 Department of Neurology, Mass General Brigham Hospital, Boston, MA, United States; 2 Department of Neurology, Universidade de São Paulo, São Paulo, Brazil; 3 Instituto de Ensino e Pesquisa, Hospital Sirio-Libanês, São Paulo, Brazil; 4 Independent Researcher, Pittsford, New York, NY, United States; 5 Department of Genetics, University of Rochester Medical Center, Rochester, NY, United States; 6 Department of Clinical Neurosciences, University of Calgary, Calgary, AB, Canada; 7 Mendelics Genomic Analysis, São Paulo, Brazil; 8 Department of Neurology, Universidade Estadual de Campinas, São Paulo, Brazil; 9 Queen Square Brain Bank for Neurological Disorders, UCL Institute of Neurology, London, United Kingdom

**Keywords:** episodic ataxia (EA), episodic ataxia 2, genetic, low resource areas, paroxysmal movement disorder

## Abstract

**Background:**

Episodic ataxias (EA) comprise a heterogeneous group of genetic conditions with spells of gait difficulty and imbalance, for which the main causes are EA1 (*KCNA1* gene) and EA2 (*CACNA1A* gene). While EA1 may respond to some antiepileptics and EA2 responds to acetazolamide, no guideline exists to inform decision-making in settings where genetic testing is unavailable.

**Objectives:**

We sought to determine distinguishing clinical features between EA1 and EA2 and propose an algorithm based on our findings.

**Methods:**

Systematized literature review to identify individuals with confirmed pathogenic variants in *KCNA1* and *CACNA1A,* followed by statistical analysis to compose a management algorithm. Subsequently, the algorithm was tested in cases described within the last three years.

**Results:**

Attack duration with a cut-off of < 10 min had high sensitivity (75.3%) and specificity (94.0%) for EA1. Additional features with high specificity included symptoms during the attacks (e.g., headaches in EA2, 95.7%) and symptoms between attacks (e.g., myokymia in EA1 99.6%; nystagmus in EA2, 98.8%). Kinesigenic triggers were more frequently reported in EA1 (68.4% vs. 5.3%, *p* < 0.001). EA1 subjects also had more frequent attacks (Daily 37.9% vs. 15.9%, *p* < 0.001) and had a lower age of onset (7y, IQR [4–10] vs. 10y, IQR [5–15], *p* = 0.003). Testing our algorithm in a case cohort yielded a sensitivity of 87.5% in identifying EA2 cases.

**Conclusion:**

EA1 and EA2 patients represent clinically different populations. We propose a management algorithm based on features with highest diagnostic accuracy, which may inform decision-making in resource-limited settings.

## Introduction

Episodic ataxias (EA) comprise a heterogeneous group of conditions characterized by spells of gait difficulty and imbalance. These can be divided into primary – where a genetic cause is known or presumed – and secondary, when it occurs as a manifestation of structural or metabolic insults to the central nervous system.

According to the Online Mendelian Inheritance in Man (OMIM) database, nine subtypes of primary episodic ataxia have been described historically and confirmed causative genes have been identified for a subset: EA1 (*KCNA1*), EA2 (*CACNA1A*), EA5 (*CACNB4*), EA6 (*SLC1A3*), and EA9 (*SCN2A*) (1–4). This list of episodic ataxia genes is not exhaustive, as additional genes have been implicated in paroxysmal ataxia (e.g., *FGF14*) but have not been consensually attributed to a numerical entry into OMIM’s episodic ataxia phenotypic series. Furthermore, other genetically determined conditions have had episodic ataxia described as possible manifestations, usually in the context of other central nervous system symptoms and mostly within the spectrum of neurometabolic conditions. This is the case of Glut-1 deficiency, pyruvate dehydrogenase deficiency, Hartnup disease, maple syrup urine disease, mitochondrial disease and certain defects in biopterin synthesis or the urea cycle. In addition to metabolic causes, episodic ataxia may also result from injury to the brain from structural or inflammatory injury. So-called secondary causes of episodic ataxia include demyelinating disease, stroke, Behçet’s disease or other auto-immune diseases such as brainstem encephalitis and certain paraneoplastic conditions (5). Finally, spells of gait difficulty may also occur in conditions with vestibular dysfunction, such as vestibular migraine or recurrent vertigo of childhood.

When the history and clinical examination suggest the possibility of a genetic episodic ataxia, EA1 and EA2 are the most frequently encountered subtypes. EA1 is caused by pathogenic variants in the *KCNA1* gene, which encodes the Kv1.1 subunit of a potassium channel (1). Disease is typically caused by missense variants that lead to decreased channel function (6). EA2 is associated with pathogenic variants in *CACNA1A* gene, which encodes the Cav2.1 subunit of a calcium channel (3, 4, 7, 8). Pathogenic variants in *CACNA1A* are also associated with other phenotypes, such as familial hemiplegic migraine 1 (FMH1), epilepsy and spinocerebellar ataxia type 6 (SCA6). Generally, EA2 is caused by loss-of-function variants (truncating or missense variants that negatively impact channel function) while FHM1 is caused by gain-of-function variants, albeit phenotypic overlap between conditions and variable expressivity have been described (9–11). In the case of SCA6, the mechanism is distinct. It is caused by a pathological repeat expansion of a trinucleotide sequence in the coding region of *CACNA1A*, generally with onset in adulthood (12).

The distinction between EA1 and EA2 is relevant. While EA1 may respond to voltage-sensitive sodium channel blockers (e.g., carbamazepine, phenytoin, lamotrigine), EA2 can ameliorate with acetazolamide or 4-aminopyridine (13, 14). Acetazolamide may also be used in EA1, but its efficacy may decrease over time and novel agents are being investigated (15). Symptom overlap between the two syndromes may preclude the formulation of a confident clinical diagnosis. We sought to analyze phenotypic information from published cases, ascertaining predictive diagnostic characteristics and creating a statistically based management algorithm with high specificity and good accuracy. Subsequently, we tested our model in a case cohort. The overarching goal was to assist clinicians in making a presumptive diagnosis when suspicion is high for EA1 or EA2, but testing is difficult or unavailable. Our work suggests that it is possible to clinically differentiate these conditions based on careful phenotyping.

## Methods

We conducted a systematic literature review in the Medline database (PubMed/NIH) and selected papers from 1993 to 2022 reporting individuals with episodic ataxia with detailed clinical and genetic data. Data from each individual reported was extracted in standardized fashion, and this was used as the “core” data set. This was used for statistical analyses, genotype–phenotype correlations and as the core dataset for building the algorithm. Subsequently, we performed a second literature search and included only papers published between 2022 and 2025. The patient data extracted from this second search comprised the “test cohort” data set. The selection criteria for the “test cohort” dataset were the same as the “core” dataset, and it required that clinical variables selected to compose the algorithm were available in detail for analysis (e.g., attack duration).

**Figure 1:**
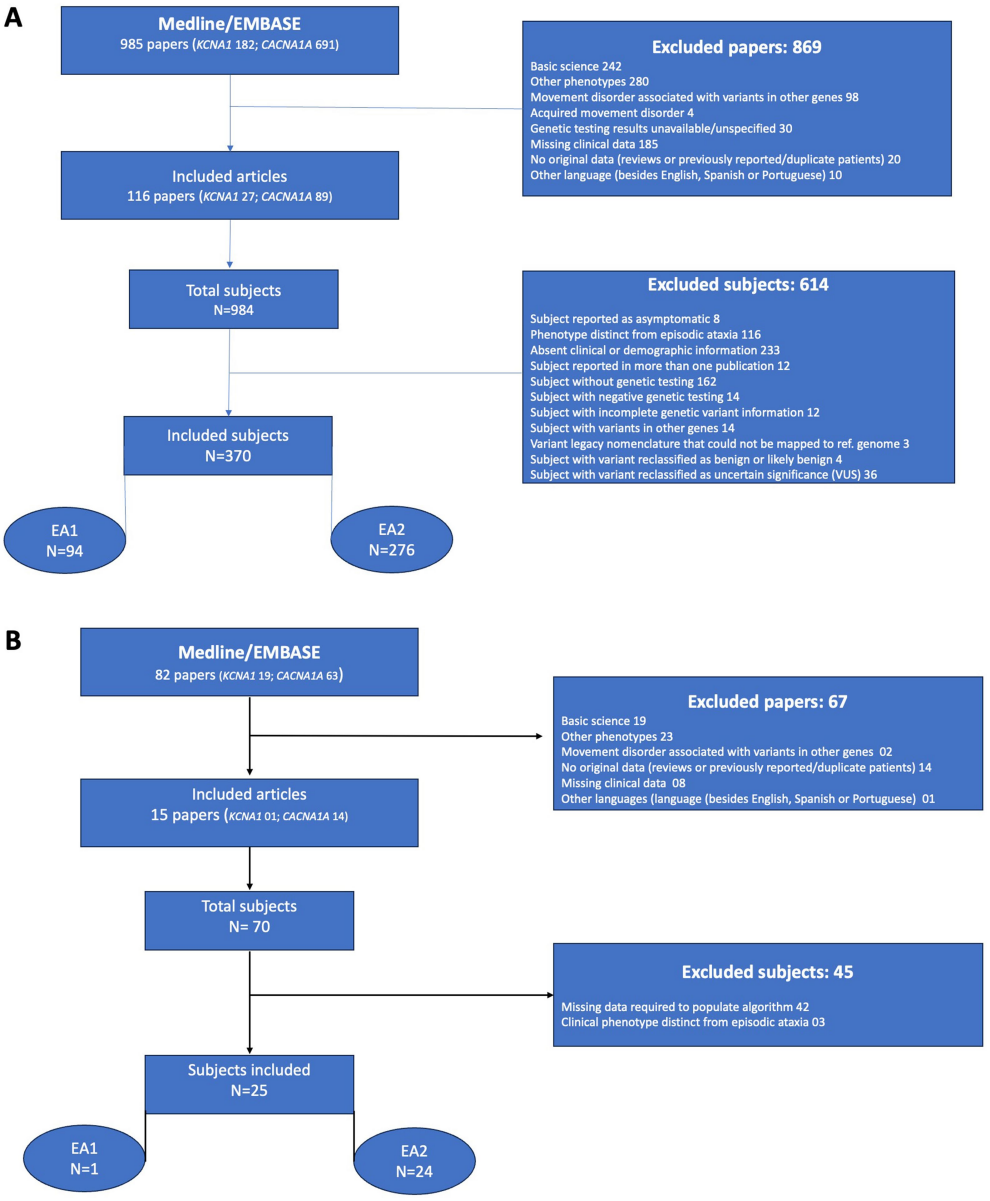
**(A)** PRISMA diagram demonstrating article selection and subject tabulation for the core dataset. **(B)** PRISMA diagram demonstrating article selection and subject tabulation for the test cohort dataset.

In order to obtain reliable clinical and genetic data we excluded papers that had alternative clinical presentations without mention of episodic ataxia, cases with trinucleotide repeat expansions in *CACNA1A,* if clinical data was grouped preventing extraction from individual cases, or if genetic testing results were incomplete or undisclosed. All genetic variants were reviewed and whenever necessary reclassified, as described below. PRISMA flow diagrams for the core and test cohort datasets can be seen in Figure 1. Details on the literature search, selection criteria and a list of recovered articles for each dataset is presented on Supplementary material S1.

Based on patterns that emerged from data analysis and specialist discussion, we constructed an algorithm to assist in the diagnosis and management of suspected EA cases. Notably, we used a systematized method to review the literature, but not a systematized method to construct the algorithm. Similar to most published management flowcharts in rare diseases, our flowchart was constructed based on specialist opinion - with the addition that, unlike most published flowcharts, the choice of clinical features used to guide management was guided by the combination of statistical evidence that emerged when analyzing the systematized review, through the selection of features with significant statistical differences between groups. We then performed a testing of this algorithm by applying it to further cases collected from the literature.

### Clinical variables

Variables collected included age of onset, sex, presence of aura, triggers, frequency, and duration of attacks and symptoms (during an attack and between episodes). The list of specific symptoms collected can be seen in Supplementary material S3. We classified triggers into kinesigenic, exercise-induced and non-kinesigenic subtypes. In the non-kinesigenic category, we further subdivided into alcohol, tobacco, sleep deprivation, physiological stress (fever/illness/infection), fatigue, fasting, caffeine, startle, heat, anxiety, menstruation, excitement and “other”(i.e., not represented in any of previously listed categories).

When data was provided as a range (e.g., duration) we included the longest/largest value, or if provided as a mean, the mean value only was included. To facilitate statistical analysis, select data was subdivided in ordinal categories, such as attack frequency (daily > = 1/day; weekly > = 1/week and < 1/day, monthly > = 1/month and < 1/week and rare/sporadic <1/month) and attack duration (brief attacks <= 10 min duration, intermediate as >10 min and <= 60 min, prolonged > 1 h and < 1 day and protracted as > = 1 day). Continuous (e.g., age of onset) and categorical (e.g., presence/absence of a symptom) data were analyzed as appropriate.

### Genetic data

We undertook legacy genetic data annotation in order to standardize our analysis. Firstly, all genetic variants were mapped to current consensus coding sequence transcripts on *KCNA1* (NM_000217.3) and *CACNA1A* (NM_001127221.2) using the GRCh38/hg38 build. Secondly, all variants were scrutinized and reassessed for pathogenicity using the widely accepted 5-tiered system recommended by the American College of Medical Genetics (16, 17). After reassessment, all subjects with non-diagnostic variants were excluded (comprising those which after reclassification had benign, likely benign or variants of uncertain significance, as well whenever legacy data did not allow proper mapping to the current genome build). Pursuant to reclassification, we then categorized variants according to mutational effect (e.g., missense, nonsense, frameshift, splicing, in-frame or out-of-frame deletions, etc.) and further subclassified variants into loss-of-function (LoF), gain of function (GoF) or unknown effect. In the LoF group, we included variants leading to haploinsufficiency (i.e., truncating variants predicted to lead to non-sense mediated decay such as certain nonsense, frameshift and out-of-frame deletions as well as splicing mutations predicted to lead to a premature stop). The LoF group also included any variant type in which functional studies were available determining a deleterious effect on channel function. Missense variants with dominant-negative effects were noted, but for functional analyses were lumped into the LoF group. The GoF group comprised variants in which functional studies were available determining an increase in channel function. Variants in the “unknown” group were predicted to be translated (e.g., missense, truncating variants not expected to lead to NMD, in-frame deletions or exon skipping in-frame splicing variants) but functional studies were unavailable to determine their impact conclusively.

Furthermore, we sought to determine if variant effect had a correlation with symptom presentation. Since for both phenotypes of interest the majority of variants identified had loss-of-function effects, we attempted to determine if variants leading to haploinsufficiency (i.e., truncating) were different than missense variants.

### Statistical analysis

We used the SPSS software package v29.0, assuming 5% significance level. We used Pearson Chi-square/Fisher for categorical variables and T-Test or Mann–Whitney for scale variables, according to normal vs. non-parametric distribution. Sensitivity, specificity and accuracy of each clinical sign extracted from the “core” dataset was calculated and used for the selection of clinical variables to differentiate EA1 and EA2. In order to create the management algorithm, we used ROC curve coordinates and listed features that had statistical significance and area under the curve (AUC) higher than 0.5. Subsequently, we refined the list to include only features that yielded specificity >90% for either condition. Finally, we further restricted the clinical variables in the algorithm to minimize redundancy and ensure reliability from extracted data. As illustrative examples, fatigue and gastrointestinal symptoms were statistically more prevalent as an attack feature in the EA2 cohort. However, the former is very subjective and the latter may mean different things (nausea, vomiting, abdominal discomfort, diarrhea, etc). This lack of precise definition and heterogeneity would ultimately be less helpful for clinicians, and through consensus these features were removed from the algorithm.

## Results

The “core” dataset search recovered 985 abstracts dating from 1994 to 2022. Subsequent screening led to the initial exclusion of 869 papers, yielding 116 papers for detailed scrutiny. Subsequently, 614 subjects were removed based on additional criteria. In total, 370 individuals with pathogenic or likely pathogenic variants were included in the final analysis (EA1 = 94; EA2 = 276; Figure 1A). Table 1 has demographic characteristics on recovered subjects.

**Table 1:** Key clinical and demographic characteristics (core dataset).

	EA1	EA2	*p* value
Subjects (*N*)	94	276	n/a
Female sex (*N*, %)	58 (61.7%)	111 (44%)	0.003^$^
Age of onset, Y (Median, 25th-75th IQR, [Range])	7.0, 4–10, [1–26]	10.0, 5–15 [0.25–61]	0.003^&^
Age at publication, Y (Mean ± SD)	34.11 ± 18.17	35.46 ± 18.17	0.558^#^
Attack duration (*N*, %)			
Brief (<= 10 min)	61 (75.3%)	11 (6.0%)	<0.001^$^
Intermediate (>10 and <= 60 min)	9 (11.1%)	33 (18.0%)	0.156^$^
Prolonged (> 60 min and < 1 day)	9 (11.1%)	119 (65.0%)	<0.001^$^
Protracted (> = 1 day)	2 (2.5%)	20 (10.9%)	0.022^$^
Subjects with missing attack duration data	22	93	n/a
Attack frequency (*N*, %)^$^			
Daily	25 (37.9%)	22 (15.9%)	<0.001^$^
Weekly	25 (37.9%)	72 (52.2%)	0.056^$^
Monthly	7 (10.6%)	34 (24.6%)	0.019^$^
Rare/sporadic	9 (13.6%)	10 (7.2%)	0.142^$^
Subjects with missing attack frequency data	28	138	n/a

^$^Chi Squared; ^&^Mann–Whitney; ^#^T-test.

The literature search for the “test cohort” dataset recovered 82 papers from 2022 through 2025, with subsequent screening leading to the exclusion of 67 papers yielding 15 papers for detailed scrutiny. This initially identified 70 subjects with pathogenic or likely pathogenic variants in *KCNA1* or *CACNA1A,* and further application of exclusion criteria removed 45 subjects, with final inclusion of 25 individuals in the test cohort dataset (EA1 = 1; EA2 = 24; Figure 1B).

## Genetic architecture (core dataset)

**Figure 2:**
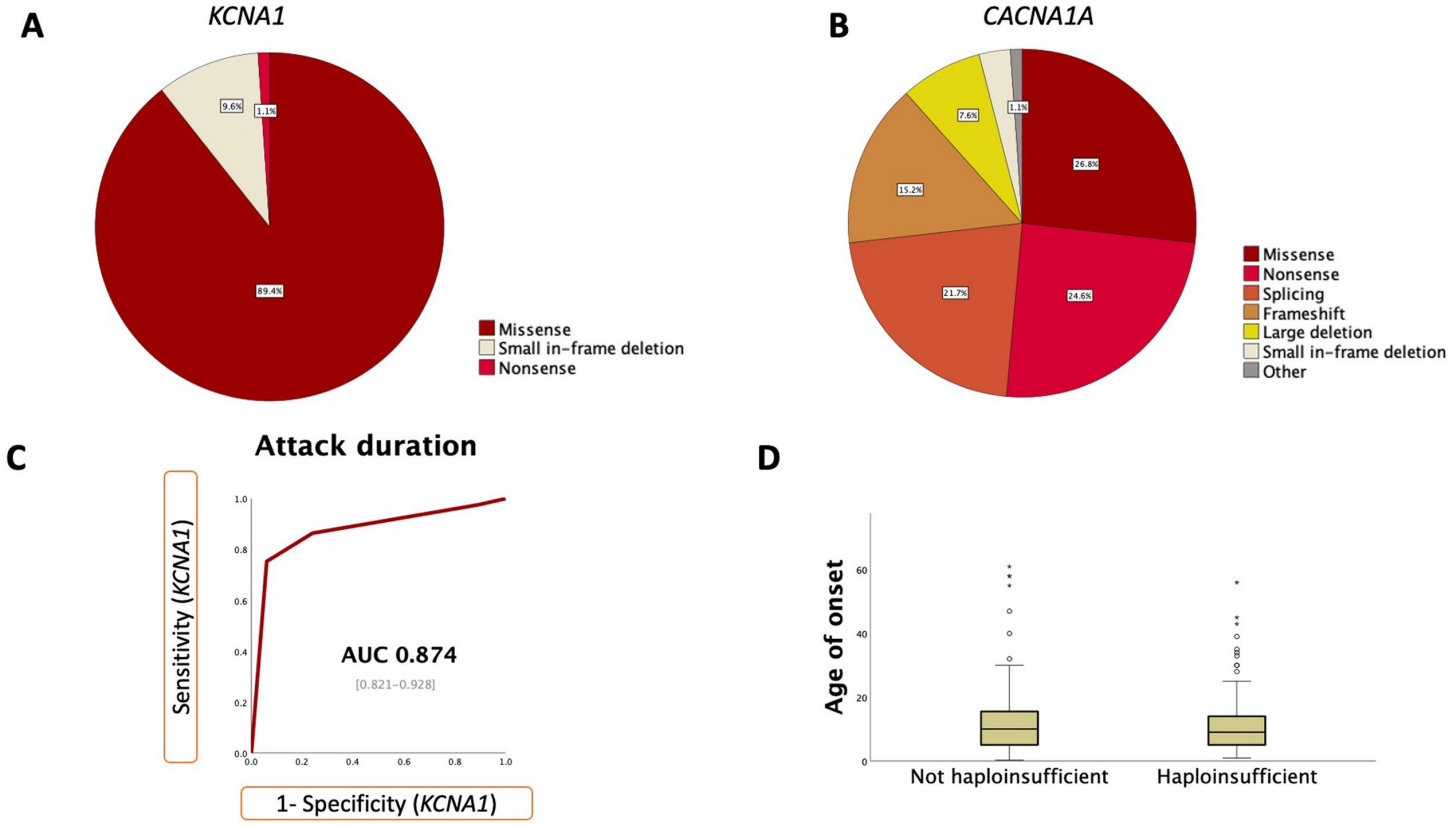
**(A)** Variant type distribution in *KCNA1*; **(B)** Variant type distribution in *CACNA1A*; **(C)** ROC curves for clinical features helpful when diagnosing EA1/*KCNA1*. Numbers represent 95% confidence interval; **(D)** Boxplot comparing age of onset in haploinsufficient vs. non-haploinsufficient variants in subjects with EA2/*CACNA1A*. ROC = receiver operator curve. AUC = area under the curve.

There were 26 distinct variants in *KCNA1*. Variant distribution can be seen in Figure 2. Details regarding variant distribution and functional effect is outlined in Supplementary materials 2.

## Clinical characteristics (core dataset)

### Gender and age of onset

In the “core” dataset, there were statistically significant differences in gender (females in EA1 61.7% vs. 44% in EA2, *p* = 0.003, Chi Squared) and age of onset (EA1 median 7 years old; IQR [4–10] vs. EA2 median 10 years old; IQR [5–15], p = 0.003, Mann–Whitney) (Table 1).

In the “test cohort,” we did not perform statistical analysis in light of the restricted number of EA1 cases recovered. In this subset, the median age of onset of EA2 was 10 years old (IQR [5–13]; range 0.1–55 years old) and 50% of subjects were of the female sex (Supplementary material S3).

### Attack duration and frequency

In the “core dataset,” individuals with EA1 had shorter attack duration. 75.3% of EA1 individuals had brief attacks, vs. 6.0% in EA2 (*p* < 0.001; Table 1). Conversely, subjects with EA2 were more likely to have prolonged (65% EA2 vs. 11.1% in EA1, *p* < 0.001) and protracted attacks (10.9% EA2 vs. 2.5% EA1, *p* = 0.022). Subjects with EA1 also had more frequent attacks (Daily attacks in EA1 37.9% vs. EA2 15.9%, *p* < 0.001; Monthly attacks in EA2 24.6% vs. EA 10.6%, *p* = 0.019).

In the “test cohort,” we did not perform statistical analysis in light of the restricted number of EA1 cases recovered. In this subset, none of the EA2 subjects had brief attacks (0/24), with most having either prolonged (12/24, 50%) or protracted attacks (3/24, 12.5%). Attack frequency was more distributed, with most EA2 individuals having either weekly (10/24, 43.4%) or monthly (9/24, 39.1%) (Supplementary material S3).

### Triggers

Trigger data was available in 193/370 subjects (52.2%). This comprised 79/94 (84%) of individuals with EA1 and 114/276 (58.6%) of individuals with EA2. When trigger data was present, it was more frequently observed in EA1 (79/79 or 100% EA1 subjects had reported triggers vs. 104/114 or 91.2% in EA2, *p* = 0.006; Fisher).

Whenever EA1 subjects had trigger data available, these were more likely to be kinesigenic (54/79 or 68.4% vs. 6/115 or 5.3%, *p* < 0.001, Fisher); non-kinesigenic (71/79 or 89.9% vs. 83/114 or 72.8%, *p* = 0.004, Chi Squared); physiological stress (35/73 or 47.9% vs. 23/110 or 20.9%, *p* < 0.001, Chi Squared); caffeine (11/77 or 14.3% vs. 5/114 or 4.4%, *p* = 0.030, Fisher); startle (39/77 or 49.4% vs. 0/114 or 0%; *p* < 0.001; Fisher), menstruation (6/77 or 7.8% vs. 0/114 or 0%, *p* = 0.004, Fisher); anxiety (20/77 or 26% vs. 9/114 or 7.9%; *p* < 0.001 Fisher). Conversely, heat was a more common trigger in EA2 (14/114 or 12.3% vs. 2/77 or 2.6% in EA1; *p* = 0.018; Fisher). There were no statistical differences between groups regarding other triggers such as exercise, emotional stress, alcohol, tobacco, sleep deprivation, fatigue or excitement.

### Attack symptoms

During an attack, individuals with EA1 were significantly more likely to report ataxia (limb and axial), rigidity and myokymia, whereas subjects with EA2 had more vertigo, gastrointestinal symptoms, headaches and fatigue (Supplementary material S3).

### Symptoms between attacks

Between attacks, EA1 subjects were significantly more likely to harbor peripheral muscular symptoms (including neuromyotonia and myokymia) and tremors, whereas EA2 subjects had more interictal ataxic symptoms, nystagmus and headaches. Ataxia was more likely to be progressive in EA2 (Supplementary material S3).

### Sensitivity and specificity analyses

We ran sensitivity and specificity analyses on the most frequently reported attack variables (e.g., duration, frequency), symptoms, triggers, features between attacks and response to medications. Table 2 reports accuracy analyses for signs which performed statistically better than a random classifier. This was determined by plotting the receiver operating characteristic (ROC) curve and ensuring that the 95% confidence interval of the area under the curve (AUC) did not overlap with 0.5. We plotted ROC curves for attack duration, age of onset, attack frequency and acetazolamide responsiveness (complete, partial or no response). Remarkably, attack duration had excellent discriminatory accuracy, with an AUC of 0.88 (95% CI 0.822–0.929; Figure 2; Supplementary material S4).

**Table 2:** Sensitivity and specificity analysis.

Variable	Gene	Sensitivity (%)	Specificity (%)	AUC	95% CI	*p* value
Attack triggers						
Kinesigenic *N* = 193	*KCNA1*	68.4	94.7	0.815	0.748–0.883	<0.001
Startle *N* = 191	*KCNA1*	49.4	100	0.747	0.669–0.824	<0.001
Non-kinesigenic *N* = 193	*KCNA1*	89.9	27.2	0.585	0.505–0.666	0.006
Physiological stress *N* = 183	*KCNA1*	47.9	79.1	0.635	0.551–0.719	<0.001
Anxiety *N* = 191	*KCNA1*	26.0	92.1	0.590	0.506–0.675	<0.001
Attack frequency and duration						
Brief attacks *n* = 264	*KCNA1*	75.3	94.0	0.846	0.787–0.906	<0.001
Daily attacks *N* = 204	*KCNA1*	37.9%	84.1%	0.610	0.525–0.696	< 0.001
Features during attack						
Limb ataxia *N* = 315	*KCNA1*	48.9	88.7	0.688	0.619–0.757	<0.001
xial ataxia *N* = 315	*KCNA1*	68.1	58.8	0.635	0.568–0.701	<0.001
Rigidity / Stiffness *N* = 314	*KCNA1*	26.6	99.5	0.631	0.558–0.703	<0.001
Myokymia *N* = 314	*KCNA1*	23.4	99.5	0.615	0.542–0.688	<0.001
Vertigo *N* = 313	*CACNA1A*	63.2	76.3	0.698	0.635–0.760	<0.001
Headache *N* = 314	*CACNA1A*	25.0	95.7	0.604	0.540–0.668	<0.001
GI symptoms *N* = 314	*CACNA1A*	44.5	91.5	0.680	0.620–0.740	<0.001
Features between attacks						
Any ataxia *N* = 326	*CACNA1A*	43.1	91.2	0.672	0.610–0.733	<0.001
Nystagmus *N* = 326	*CACNA1A*	63.4	98.8	0.811	0.765–0.856	<0.001
Clinical myokymia *N* = 326	*KCNA1*	40.0	99.6	0.698	0.622–0.774	<0.001
Neuromyotonia *N* = 326	*KCNA1*	20.0	100.0	0.600	0.523–0.677	<0.001
EMG myokymia *N* = 325	*KCNA1*	48.1	100.0	0.741	0.666–0.815	<0.001
Peripheral muscular symptoms *N* = 326	*KCNA1*	82.5	99.2	0.908	0.858–0.958	<0.001

Overall, reported symptoms (either during or between attacks) had low sensitivity but good specificity. For example, the presence of clinical myokymia between attacks (a symptom typically attributed to EA1) had sensitivity of 40% and specificity of 99.6%, while headaches during an attack had a sensitivity of 25% but specificity of 95.7% for EA2. Notable features with higher sensitivity included the presence of triggers (kinesigenic and non-kinesigenic) in EA1 and vertigo during attacks in EA2.

### Treatment response

Drug treatment and response were inconsistently reported. When data was available, symptom response was more likely with acetazolamide in EA2 (complete or partial remission in 139/162 or 85.8% vs. 17/33 or 51.5%, *p* < 0.001; Table 3).

**Table 3:** Drug treatment.

Drug	*CACNA1A* Absolute freq	*CACNA1A* %	*KCNA1* Absolute freq	*KCNA1*%	*p* value**
Mentions drug treatment	174/192	90.3%	54/65	83.1%	0.096
Any drug response *	155/173	89.6%	28/54	51.9%	<0.001
Acetazolamide response	139/162	85.8%	17/33	51.5%	<0.001
Phenytoin response	1/3	33.3%	4/6	66.7%	0.343
Carbamazepine response	3/9	33.3%	4/19	21.1%	0.483
4AP response	11/11	100%	n/a	n/a	n/a
Pyridostigmine response	1/1	100%	n/a	n/a	n/a
Flunarizine response	4/4	100%	n/a	n/a	n/a
Valproic acid response	1/3	33%	1/3	33.3%	1.000
Topiramate response	1/1	100%	0/1	0%	0.157

*Aggregate data. Response was defined as partial or complete improvement.

**Chi Squared.

### Genotype–phenotype correlation

In *CACNA1A*, individuals with non-haploinsufficient variants were more likely to have headaches (15/78 or 19.2% vs. 17/168 or 10.1%; *p* = 0.048; Chi Squared); progressive ataxia (12/58 or 20.7% vs. 7/115 or 6.1%; Chi Squared *p* = 0.004), autonomic symptoms during the attack (7/84 or 8.3% vs. 1/136 or 0.7%; Chi Squared *p* = 0.006) and cerebellar atrophy (52.6% or 20/38 vs. 23.5% or 18/76; Chi Squared *p* = 0.002). There were no statistically significant differences in age of onset (Figure 2), attack frequency, attack duration or other clinical features during or between attacks. In *KCNA1*, we were not able to compare clinical variables with functional effect as most variants were missense.

### Management algorithm and performance

**Figure 3:**
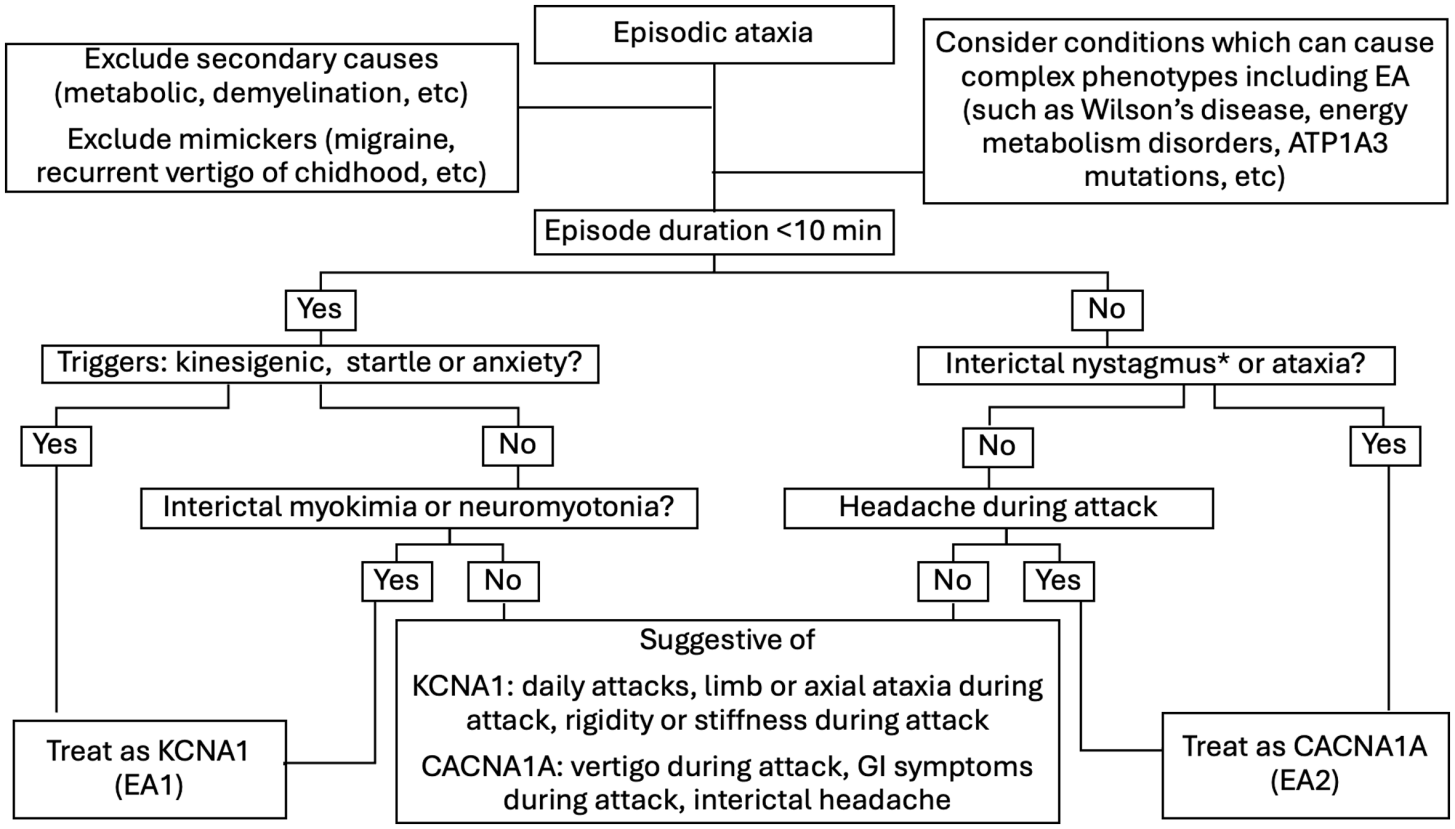
Proposed management algorithm based on clinical features to differentiate EA1 vs. EA2. * While our model calculated data when/if cases had manifestations of nystagmus between attacks, in clinical practice EA2 individuals may more broadly demonstrate interictal cerebellar oculomotor disorders.

Based on criteria established in the methodology section and our results, we created a management algorithm designed for use in limited resource settings in which genetic testing is unavailable (Figure 3). Applying our proposed algorithm on the test cohort yielded a sensitivity of 87.5% in identifying EA2 cases. For reference, a flowchart with the algorithm populated with cases from the test cohort dataset can be seen in Supplementary material S5.

## Discussion

Our results show that there are statistically significant differences in the clinical presentation of EA1 vs. EA2. EA1 is more common in females and has an earlier age of onset, with shorter lasting and more frequent attacks. Attack duration, with an optimal cut-off at 10 min, had 75.3% sensitivity and 94.4% specificity for EA1. Triggers with high specificity for EA1 include startle and movement initiation (kinesigenic), a finding that we previously reported (15). Prototypically, interictal peripheral neuromuscular symptoms are common in EA1 while interictal cerebellar dysfunction is seen in EA2 (see Supplementary Video). In addition to confirming these findings, we found other symptoms with excellent specificity when present, such as anxiety as a trigger (EA1), headache during an attack (EA2) and GI symptoms during an attack (EA2).

Applying features with high specificity in sequential fashion through our proposed algorithm yielded a sensitivity of 87.5 and 100% specificity for the diagnosis of *CACNA1A* in the test cohort datase. Considering that EA2 can be clinically successfully managed with acetazolamide and/or 4-aminopyridine (unlike EA1-*KCNA1*) and that in limited resource settings the management of EA is typically empirical, our results provide applicable evidence for clinicians facing these patients in this context.

We also looked at statistically significant differences in therapeutic response to prescribed drugs. Numbers were limited for several agents, but in a substantial proportion of individuals, data were available regarding acetazolamide response. Our findings confirm that EA2 subjects are much more likely to respond to acetazolamide than EA1 subjects (Table 3). This finding could lead to the inference that a therapeutic trial with acetazolamide could help differentiate one condition from the other, as opposed to going through our proposed algorithm. We advise against using a therapeutic trial to differentiate these conditions. In this retrospective analysis of published cases, we cannot determine whether genotype may have influenced prescription patterns and potentially skewed data interpretation. Therefore, we believe that estimating sensitivity and specificity of acetazolamide response in this scenario may not be reflective of real practice, where genotype is unknown. Such analysis is more likely to be accurate if done in prospective fashion. Furthermore, we would like to point out that 4-aminopyridine is also an effective treatment for EA2, as previously reported in the literature (18–20) and as summarized in Table 3, it was 100% effective (11/11 patients reported).

The vast majority of individuals with EA2 had LoF variants. Some differences emerged when comparing phenotypes of individuals with variants leading to haploinsufficiency vs. non-haploinsufficient variants. Individuals with non-haploinsufficient variants were more likely to have progressive ataxia and cerebellar atrophy. These findings were unexpected and may contribute to the understanding of the underlying pathophysiology in *CACNA1A*-related phenotypes. Both haploinsufficiency and dominant negative are known disease mechanisms in EA2 (21). It is possible to conjecture that expressing a dysfunctional CACNA1A protein may be more toxic to the cerebellum than having decreased expression. In the neurodegenerative condition SCA6, a polyglutamine expansion in the *CACNA1A* protein leads to cerebellar toxicity. It should be noted however that *CACNA1A* undergoes extensive alternative splicing, therefore variant effect prediction in tissue-specific fashion is a complex task (22). Further research on molecular mechanisms subserving channel dysfunction pertaining to specific variants could be informative of genotype–phenotype correlations in EA2.

Our study has several limitations. We have conceived our clinical algorithm to be most helpful for clinicians facing patients with early (i.e., childhood-onset) episodic ataxia in low-resource settings. Therefore, the context matters. A fundamental assumption in following this algorithm is that the treating physician has a high suspicion for EA1 and EA2 but cannot obtain molecular confirmation. This work is not an algorithm for the differential diagnosis of imbalance or vertigo in children, which would need to include clinical features to diagnose vestibular migraine (such as photophobia or phonophobia, for instance) and recurrent vertigo of childhood (RVC), both conditions which are prevalent and have appropriate treatments. Furthermore, our work is not inclusive of all episodic ataxia etiologies and therefore care should be taken when applying this flowchart. A careful exclusion of secondary causes of episodic ataxia is always mandatory. Additionally, even when suspicion for a genetic etiology is high – as in familial cases - episodic ataxia may be the manifestation of a more complex neurogenetic disorder such as Wilson’s disease and other neurometabolic syndromes, which require careful evaluation. Our algorithm only applies to patients with clinical features suggestive of genetically caused pure episodic ataxia, which comprises a particular subset of EA patients. In this group, EA2 is most prevalent, followed by EA1 (23). Therefore, when evaluating children with periodic imbalance and before using our algorithm, we strongly suggest colleagues to look for clues suggestive of alternative diagnoses, such as RVC (20), vestibular migraine (24) and other forms of hereditary episodic ataxia (10).

Furthermore, in individuals with additional neurological symptoms or systemic signs, or in those in which neuroimaging abnormalities exist, prior to following this algorithm clinicians should strongly consider alternative primary or secondary etiologies. In particular, metabolic, structural or inflammatory changes in ancillary studies (e.g., bloodwork, imaging or cerebrospinal fluid analysis when indicated) should be clues to secondary etiologies. Even if a high index of suspicion remains for a genetic etiology, there are emerging causes of genetically determined EA for which knowledge about prevalence and clinical manifestations are still evolving. Such is the case with pathogenic point mutations in *FGF14*, where recent reports indicate that some patients may have onset of episodic symptoms in childhood (25). We emphasize that the gold standard for diagnosing and managing genetically determined ataxias is through genetic testing, while acknowledging that country and population-specific disparities often lead to difficulties in obtaining testing (26, 27).

Another limitation in our study is reliance on literature reports. Furthermore, due to the heterogeneous quality of the reports, we were forced to exclude many individuals in which data was missing and/or poorly reported. This compounds the fact that the data predominantly derives from case reports and case series, which inherently lack the rigor of prospective studies. With this concern in mind, we attempted to be as rigorous as possible in that the inclusion/exclusion process occurred within pre-defined criteria, and we strictly included only individuals for which we were confident enough to use data in statistical analysis. In the construction of our algorithm, we excluded symptoms in which we did not feel there was sufficient detail in the data to allow meaningful conclusions. While these constraints were placed to enhance the quality of the study by reducing statistical noise, these imposed restrictions carry inherent risks of selection and reporting biases.

Additionally, the analysis was done in cross-sectional fashion, so any potential discerning features that could emerge with over time may have been missed. Finally, we acknowledge that results from the analysis of our test cohortmay be of limited utility since during this period only a single case of EA1 with clinical details sufficient for inclusion was published. Therefore, the most reliable measure extracted from the test cohort dataset would be the algorithm’s sensitivity for EA2 diagnosis, since this would depend exclusively on the total number of EA2 cases and the number of these in which the classification was positive; a result that is not mathematically influenced by EA1 prevalence in the cohort. Further studies in a real-world cohort are warranted and should ideally include a reasonable number of EA1 cases, which would enable estimates of overall accuracy of our proposed algorithm, as well as sensitivity and specificity to the diagnosis of both EA1 and EA2.

Notwithstanding all aforementioned limitations, to the best of our knowledge, this is the first attempt in quantifying predictive diagnostic features using published EA1 and EA2 cases and proposing an algorithm for clinical management when genetic testing is not available. In light of the limitations described above, we do not claim this to be an evidence-based recommendation. For example, while attack duration is useful in differentiating EA1 and EA2 and is featured in our algorithm, EA1 patients can have longer attack duration as previously reported in the literature (20). Instead, we hope this work may prove useful in resource-limited settings while acknowledging that it requires prospective validation. Notably, at a purely Bayesian level, EA2 is generally the most common type of EA (7). An accurate pre-test probability would have to include local prevalence rates. We therefore recommend interpreting our algorithm according to local context and using clinical judgement.

A recent genotype–phenotype correlation study in episodic ataxias was published (23). The authors described the phenotypic overlap between the conditions and noted a few distinguishing features, such as higher frequency of interictal myokymia and shorter event duration in EA1 as well as ataxia and nystagmus in EA2. Of note, in clinical practice individuals with EA2 may demonstrate not only nystagmus between attacks, but more broadly present with “interictal cerebellar oculomotor disorders. We believe our study complements these findings by attributing numerical estimates with sensitivity and specificity analysis and further expands knowledge by reporting novel findings and proposing a management algorithm based on available evidence.

In conclusion, our study may assist clinicians in detecting discerning clinical features of EA1 and EA2 and guide testing and/or management decisions in settings where resources are limited. Furthermore, this study may also inform studies aiming to unravel molecular mechanisms underlying genotype–phenotype differences in EA2. Lastly, the methodology used in this work may be considered for the study of other rare diseases, when assembling large prospective studies may be challenging due to the rarity of the conditions.

## Data Availability

The original contributions presented in the study are included in the article/Supplementary material, further inquiries can be directed to the corresponding author.

## Supplementary material

SUPPLEMENTARY VIDEO 1 22 yo male with *CACNA1A* pathogenic variant p.Arg1857*. He endorses spells of imbalance and nausea occurring approximately every 2-3 months, lasting 2 days on average. Onset occurred in the first year of life. On video, interictal examination demonstrates bilateral intention tremor with slight dysmetria, esotropia, horizontal nystagmus and widened base. Spells have decreased in frequency after starting acetazolamide.

SUPPLEMENTARY VIDEO 2 13 yo female with *CACNA1A* pathogenic variant p.Arg1664Gln. This variant was inherited from her father who has a history of hemiplegic migraines. She has had slowly progressive ataxia since birth, without exacerbations. She endorses intermittent headaches associated with nausea and vomiting as well as learning difficulties in school. Parents report behavioral outbursts. Acetazolamide was tried but ineffective. Interictal examination on video shows esotropia, horizontal nystagmus, scanning dysarthria, dysmetria. Total SARA score: 19.

SUPPLEMENTARY VIDEO 3 13 yo male (fraternal twin of patient depicted on Supplementary Video 2), with *CACNA1A* pathogenic variant p.Arg1664Gln. He has had slowly progressive ataxia without episodic exacerbation. Clinical examination on video demonstrates esotropia, horizontal nystagmus, slight dysarthria and dysmetria more prominent in the lower extremities. Total SARA score: 14.

SUPPLEMENTARY VIDEO 4 6yo female with *CACNA1A* pathogenic variant p.Arg1664Gln. She is the sibling of patients shown on Supplementary Videos 2, 3. She has more pronounced chronically progressive ataxia with learning disability. Video shows apparent oculomotor apraxia with saccade initiation, dysarthric and limited speech, significant dysmetria and truncal ataxia requiring one person assist. Total SARA score 25, 5.
