# Supplementary material for: Diagnostic approach to episodic ataxia types 1 and 2: a proposed algorithm for limited resource-settings
Source: Front Neurol. 2026 Apr 21;17:1735246. doi: 10.3389/fneur.2026.1735246 (PMC13141855; doi:10.3389/fneur.2026.1735246)
Supplement: Supplementary file 1 [file Supplementary_file_1.docx]

Supplementary Material – S1

**Information sources and search strategy:**

Core dataset:

We searched PubMed using the following search strings, with results limited from 1993 through 07/31/2022.

*Episodic Ataxia type 1:*

(((((((((potassium voltage-gated channel subfamily A member 1) OR (AEMK)) OR (KCNA1)) OR (Kv1.1)) OR (RBK1)) OR (HUK1)) OR (Episodic ataxia type 1)) OR (Episodic ataxia with myokymia)) AND (Ataxia))

*Episodic Ataxia type 2:*

(((((((((("Episodic ataxia type 2") OR (EA2)) OR (FHM)) OR (CACNL1A4)) OR (CACNA1A)) OR (FHM)) OR (Cav2.1)) OR (calcium channel, voltage-dependent, P/Q type, alpha 1A subunit)) OR (MHP)) OR (MHP)) AND (ataxia)

Validation dataset:

We searched PubMed using the following search strings, limiting results from 08/1/22 through 6/10/2025:

*Episodic Ataxia type 1:*

(((((((((potassium voltage-gated channel subfamily A member 1) OR (AEMK)) OR (KCNA1)) OR (Kv1.1)) OR (RBK1)) OR (HUK1)) OR (Episodic ataxia type 1)) OR (Episodic ataxia with myokymia)) AND (Ataxia))

*Episodic Ataxia type 2:*

(((((((((("Episodic ataxia type 2") OR (EA2)) OR (FHM)) OR (CACNL1A4)) OR (CACNA1A)) OR (FHM)) OR (Cav2.1)) OR (calcium channel, voltage-dependent, P/Q type, alpha 1A subunit)) OR (MHP)) OR (MHP)) AND (ataxia)

**Eligibility Criteria:**

Only papers that had clinical information and detailed genetic data including variant type, chromosomal position, coding and/or protein change were included. We excluded papers that had alternative clinical presentations without mention of episodic ataxia (e.g., spinocerebellar ataxia or familial hemiplegic migraine in *CACNA1A*), individuals with trinucleotide repeat expansions in *CACNA1A*, if clinical data was vague or if genetic testing results were incomplete, undisclosed or could not be confidently mapped to hg38 genome build.

**Study selection and record management**

Author LHMR Garcia identified papers, reviewed title/abstracts to determine whether they met inclusion or exclusion criteria and entered them in a spreadsheet. Subsequently, attempts were made to retrieve the full texts of all potentially relevant records. Retrieved full texts were then made available for review for three authors (LHMR Garcia, CM de Gusmao and L Silveira-Moriyama). Author CM de Gusmao reviewed individual patient data. Disagreements were resolved by consensus.

**Data collection**

Three authors (LHMR Garcia, CM de Gusmao and L Silveira-Moriyama) read published reviews on EA1 and EA2, and agreed on a set of reference, demographic and clinical variables of interest for the manuscript. These variables included the year the paper was published, journal, first and last author, subject age of onset, subject age when the article was published, sex, genetic variant as reported in the paper, presence of aura, triggers, frequency and duration of attacks, interictal manifestations, attack features, ancillary investigations and treatment response to different drugs. When data was provided as a range (e.g., duration) we included the longest time, or if provided as a mean, the mean value only was included. We consensually agreed to categorize attack duration and attack frequency in ordinal classification (e.g. attack frequency: daily >=1/day; weekly >=1/week and < 1/day, monthly >=1/month and < 1/week and rare/sporadic <1/month; attack duration: brief attacks <= 10 min duration, intermediate as >10 min and <= 60 min, prolonged > 1 hour and < 1 day and protracted as >= 1 day). Subsequently, a template data extraction spreadsheet was used to extract relevant data with a coding system for uniform data categorization. This data was extracted and entered by LHMR Garcia and subsequently reviewed by CM de Gusmao. Disagreements were resolved by consensus. Individuals that were definitely or suspected to have been published more than once were then removed in this iteration. Subsequently, genetic data was annotated and reviewed. Additional fields were added including chromosomal position, coding DNA and amino acid substitution (whenever applicable), variant type and ACMG pathogenicity criteria using guidelines (see below on genetic annotation and pathogenicity scoring). Individuals with variants that were classified as variants of uncertain significance or below were excluded.

**Data Synthesis**

Data was then quantatively analyzed importing the spreadsheet contents onto IBM SPSS Statistics (v29.0)

**Genetic annotation and classification**

All variants were lifted over to human genome build GRCh38/hg38 build (<https://liftover.broadinstitute.org/> ).

Legacy data was renamed using recommendations from the Human Genome Organization (HUGO; <https://varnomen.hgvs.org/>) and mapped to consensus coding sequence transcripts on *KCNA1* (NM_000217.3) and *CACNA1A* (NM_001127221.2).

Subsequently, we classified variants according to the mutational effect into 10 possible categories: missense, frameshift, nonsense, splicing, in-frame deletion, in-frame indel, large deletion, in-frame insertion, complex rearrangement or synonymous.

In the manuscript, we collectively refer to variants leading to *haploinsufficiency* comprising all the variants in which the transcript is expected to undergo non-sense mediated decay (ie, nonsense, frameshift, splicing) together with complex rerrangements and large deletions.

Finally, we subclassified variants into functional categories, according to the availability of in vitro or in vivo studies in the medical literature. This subclassification ascribed variants into Loss of Funcion (comprising haploinsufficiency and missense variants for which a functional study was available, determining a loss of channel function or dominant negative mechanism) and Gain of Function.

**Variant pathogenicity scoring**

All variants were scrutinized and had entries crosschecked on published literature databases. Subsequently, they were score in the 5-tiered system as suggested by the American College of Medical Genetics (ACMG), using the following information:

- Determining variant position characteristics using University of California Santa Cruz genome browser (<https://genome.ucsc.edu/> )
- For null variants, ensuring that non-sense mediated decay was expected (occasionally using tools such as <https://autopvs1.bgi.com/> ; <https://nmdprediction.shinyapps.io/nmdescpredictor/>)
- Scores of in-silico prediction tools (REVEL, CADD, SpliceAI as applicable)
- Population allele frequency using Genome Aggregation Database (GnomAD, versions 2 and 3)
- Verifying entries in published literature databases, to ensure consistency of reporting and/or availability of functional studies:
  - Human Genome Mutation Database (<https://www.hgmd.cf.ac.uk/ac/index.php>)
  - Genomenon Mastermind ( <https://mastermind.genomenon.com/>)
- Cross-checking entries in variant repositories
  - NIH ClinVar (<https://www.ncbi.nlm.nih.gov/clinvar/>)
  - Leiden Open Variation Database (<https://www.lovd.nl/> ).

Only pathogenic and likely pathogenic variants were included. Individuals with variants interpreted as of uncertain significance or below, as well those who could not be lifted over due to conflicting data were excluded.

List of recovered articles – Core Dataset

## EA 1 - *KCNA1*

1. Lassche S, Lainez S, Bloem BR, van de Warrenburg BPC, Hofmeijer J, Lemmink HH, et al. A novel KCNA1 mutation causing episodic ataxia type I. Muscle Nerve. 2014 Aug 1;50(2):289–91.

2. Imbrici, Paola, et al. "A novel KCNA1 mutation identified in an Italian family affected by episodic ataxia type 1." Neuroscience 157.3 (2008): 577-587.

3. Lee H, Wang H, Jen JC, Sabatti C, Baloh RW, Nelson SF. A novel mutation in KCNA1 causes episodic ataxia without myokymia. Hum Mutat. 2004 Dec 1;24(6):536–536.

4. Zuberi SM, Eunson LH, Spauschus A, De Silva R, Tolmie J, Wood NW, et al. A novel mutation in the human voltage-gated potassium channel gene (Kv1.1) associates with episodic ataxia type 1 and sometimes with partial epilepsy. Brain. 1999 May 1;122(5):817–25.

5. Rajakulendran S, Tan SV, Matthews E, Tomlinson SE, Labrum R, Sud R, et al. A Patient with Episodic Ataxia and Paramyotonia Congenita Due to Mutations in Kcna1 and Scn4a. Neurology. 2009 Sep 22;73(12):993–5.

6. Gancher ST, Nutt JG. Autosomal dominant episodic ataxia: a heterogeneous syndrome. Mov Disord. 1986;1(4):23953.

7. Poujois A, Antoine J-C, Combes A, Touraine RL. Chronic neuromyotonia as a phenotypic variation associated with a new mutation in the KCNA1 gene. J Neurol. 2006 Jul 1;253(7):957–9.

8. Eunson LH, Rea R, Zuberi SM, Youroukos S, Panayiotopoulos CP, Liguori R, et al. Clinical, genetic, and expression studies of mutations in the potassium channel gene KCNA1 reveal new phenotypic variability. Ann Neurol. 2000 Oct 1;48(4):647–56.

9. Tomlinson, Susan Elizabeth, et al. "Clinical, genetic, neurophysiological and functional study of new mutations in episodic ataxia type 1." Journal of Neurology, Neurosurgery & Psychiatry 84.10 (2013): 1107-1112.

10. Brownstein CA, Beggs AH, Rodan L, Shi J, Towne MC, Pelletier R, et al. Clinical heterogeneity associated with KCNA1 mutations include cataplexy and nonataxic presentations. Neurogenetics. 2016 Jan 1;17(1):11–6.

11. Çomu, Sinan, Vinodh Narayanan, and Michael Giuliani. "Episodic ataxia and myokymia syndrome: a new mutation of potassium channel gene Kv1. 1." Annals of Neurology: Official Journal of the American Neurological Association and the Child Neurology Society 40.4 (1996): 684-687.

12. Browne DL, Gancher ST, Nutt JG, Brunt ER, Smith EA, Kramer P, et al. Episodic ataxia/myokymia syndrome is associated with point mutations in the human potassium channel gene, KCNA1. Nat Genet. 1994 Oct;8(2):136–40.

13. Klein, A., et al. "Episodic ataxia type 1 with distal weakness: a novel manifestation of a potassium channelopathy." Neuropediatrics 35.02 (2004): 147-149.

14. Kinali M, Jungbluth H, Eunson LH, Sewry CA, Manzur AY, Mercuri E, et al. Expanding the phenotype of potassium channelopathy: severe neuromyotonia and skeletal deformities without prominent Episodic Ataxia. Neuromuscular Disorders. 2004 Oct 1;14(10):689–93.

15. Brunt ER, van Weerden TW. Familial paroxysmal kinesigenic ataxia and continuous myokymia. Brain. 1990 Oct;113 ( Pt 5):1361–82.

16. Hereditary myokymia and paroxysmal ataxia linked to chromosome 12 is responsive to acetazolamide. - PubMed - NCBI [Internet]. [cited 2019 Mar 16]. Available from: https://www.ncbi.nlm.nih.gov/pubmed/7561920

17. Browne DL, Brunt ERP, Griggs RC, Nutt JG, Gancher ST, Smith EA, et al. Identification of two new KCNA1 mutations in episodic ataxia/myokymia families. Hum Mol Genet. 1995 Sep 1;4(9):1671–2.

18. Gilbert GJ, Graves TD, Kullmann DM. Nongenetic factors influence severity of episodic ataxia type 1 in monozygotic twins. Neurology. 2011 Feb 1;76(5):490–490.

19. Shook SJ, Mamsa H, Jen JC, Baloh RW, Zhou L. Novel mutation in KCNA1 causes episodic ataxia with paroxysmal dyspnea. Muscle Nerve. 2008 Mar 1;37(3):399–402.

20. D'Adamo, Maria C., et al. "Novel phenotype associated with a mutation in the KCNA1 (Kv1. 1) gene." Frontiers in physiology 5 (2015): 525.

21. Mestre, Tiago A., et al. "A novel KCNA1 mutation in a family with episodic ataxia and malignant hyperthermia." Neurogenetics 17.4 (2016): 245-249.

22. Imbrici, Paola, et al. "A novel KCNA1 mutation in a patient with paroxysmal ataxia, myokymia, painful contractures and metabolic dysfunctions." Molecular and Cellular Neuroscience 83 (2017): 6-12.

23. Karalok, Zeynep S., et al. "Identification of a new de novo mutation underlying regressive episodic ataxia type I." Frontiers in neurology 9 (2018): 587.

24. Yuan, Haiming, et al. "Two novel KCNA1 variants identified in two unrelated Chinese families affected by episodic ataxia type 1 and neurodevelopmental disorders." Molecular Genetics & Genomic Medicine 8.10 (2020): e1434.

###

## EA 2 – *CACNA1A*

1. Tomlinson SE, Tan SV, Burke D, Labrum RW, Haworth A, Gibbons VS, et al. In vivo impact of presynaptic calcium channel dysfunction on motor axons in episodic ataxia type 2. Brain. 2016 Feb;139(2):380–91.

2. Ophoff, Roel A., et al. "Familial hemiplegic migraine and episodic ataxia type-2 are caused by mutations in the Ca2+ channel gene CACNL1A4." Cell 87.3 (1996): 543-552.

3. Yue, Qing, et al. "De novo mutation in CACNA1A caused acetazolamide‐responsive episodic ataxia." American journal of medical genetics 77.4 (1998): 298-301.

4. Jen, J. M. D. P., G. W. Kim, and R. W. Baloh. "Clinical spectrum of episodic ataxia type 2." Neurology 62.1 (2004): 17-22.

5. Guida, Serena, et al. "Complete loss of P/Q calcium channel activity caused by a CACNA1A missense mutation carried by patients with episodic ataxia type 2." The American Journal of Human Genetics 68.3 (2001): 759-764.

6. Strupp, M., et al. "A randomized trial of 4-aminopyridine in EA2 and related familial episodic ataxias." Neurology 77.3 (2011): 269-275.

7. Shin, Kyong Jin, et al. "A novel mutation of CACNA1A gene in episodic ataxia type 2 family in Korea." Neurology Asia 19.4 (2014).

8. Magis D, Boon E, Coppola G, Daron A, Schoenen J. A novel CACNA1A mutation results in episodic ataxia with migrainous features without headache. Cephalalgia. 2012 Nov;32(15):1147–9.

9. Fujioka S, Rayaprolu S, Sundal C, Broderick DF, Langley WA, Shoffner J, et al. A novel de novo pathogenic mutation in the CACNA1A gene. Movement Disorders. 2012 Oct;27(12):1578–9.

10. Matsuyama Z, Murase M, Shimizu H, Aoki Y, Hayashi M, Hozumi I, et al. A novel insertion mutation of acetazolamide-responsive episodic ataxia in a Japanese family. Journal of the Neurological Sciences. 2003 Jun;210(1–2):91–3.

11. Jen J, Yue Q, Nelson SF, Yu H, Litt M, Nutt J, et al. A novel nonsense mutation in CACNA1A causes episodic ataxia and hemiplegia. Neurology. 1999;53(1):34–34.

12. Romaniello R, Zucca C, Tonelli A, Bonato S, Baschirotto C, Zanotta N, et al. A wide spectrum of clinical, neurophysiological and neuroradiological abnormalities in a family with a novel CACNA1A mutation. Journal of Neurology, Neurosurgery & Psychiatry. 2010 Aug 1;81(8):840–3.

13. Krishnan AV, Bostock H, Ip J, Hayes M, Watson S, Kiernan MC. Axonal function in a family with episodic ataxia type 2 due to a novel mutation. Journal of Neurology. 2008 May;255(5):750–5.

14. Roubertie A, Echenne B, Leydet J, Soete S, Krams B, Rivier F, et al. Benign paroxysmal tonic upgaze, benign paroxysmal torticollis, episodic ataxia and CACNA1A mutation in a family. Journal of neurology. 2008;255(10):1600–1602.

15. Scoggan KA, Friedman JH, Bulman DE. CACNA1A mutation in a EA-2 patient responsive to acetazolamide and valproic acid. Canadian Journal of Neurological Sciences/Journal Canadien des Sciences Neurologiques. 2006;33(01):68–72.

16. Wan J, Khanna R, Sandusky M, Papazian DM, Jen JC, Baloh RW. CACNA1A mutations causing episodic and progressive ataxia alter channel trafficking and kinetics. Neurology. 2005 Jun 28;64(12):2090–7.

17. Robbins MS, Lipton RB, Laureta EC, Grosberg BM. CACNA1A Nonsense Mutation is Associated With Basilar-Type Migraine and Episodic Ataxia Type 2. Headache: The Journal of Head and Face Pain. 2009 Jul;49(7):1042–6.

18. Yugrakh MS, Levy OA. Clinical Reasoning: A middle-aged man with episodes of gait imbalance and a newly found genetic mutation. Neurology. 2012;79(16):e135–e139.

19. Mantuano E. Clusters of non-truncating mutations of P/Q type Ca2+ channel subunit Cav2.1 causing episodic ataxia 2. Journal of Medical Genetics. 2004 Jun 1;41(6):e82–e82.

20. Sivák, Štefan, et al. "Novel missense variant of CACNA1A gene: A case report of a family with episodic ataxia type 2." Journal of the neurological sciences 376 (2017): 119-120.

21. Yue Q, Jen JC, Thwe MM, Nelson SF, Baloh RW. De novo mutation in CACNA1A caused acetazolamide-responsive episodic ataxia. Am J Med Genet. 1998 May 26;77(4):298–301.

22. Friend KL, Crimmins D, Phan TG, Sue CM, Colley A, Fung VS, et al. Detection of a novel missense mutation and second recurrent mutation in the CACNA1A gene in individuals with EA-2 and FHM. Human genetics. 1999;105(3):261–265.

23. Imbrici P. Dysfunction of the brain calcium channel CaV2.1 in absence epilepsy and episodic ataxia. Brain. 2004 Nov 10;127(12):2682–92.

24. Cricchi F, Di Lorenzo C, Grieco GS, Rengo C, Cardinale A, Racaniello M, et al. Early-onset progressive ataxia associated with the first CACNA1A mutation identified within the I?II loop. Journal of the Neurological Sciences. 2007 Mar;254(1–2):69–71.

25. Claassen J, Teufel J, Kalla R, Spiegel R, Strupp M. Effects of dalfampridine on attacks in patients with episodic ataxia type 2: an observational study. Journal of Neurology. 2013 Feb;260(2):668–9.

26. Pradotto L, Mencarelli M, Bigoni M, Milesi A, Di Blasio A, Mauro A. Episodic ataxia and SCA6 within the same family due to the D302N CACNA1A gene mutation. Journal of the Neurological Sciences. 2016 Dec;371:81–4.

27. van den Maagdenberg AMJM, Kors EE, Brunt ER, van Paesschen W, Pascual J, Ravine D, et al. Episodic ataxia type 2. Journal of Neurology. 2002 Nov 1;249(11):1515–9.

28. Kim J-M, Kim JS, Ki C-S, Jeon B-S. Episodic ataxia type 2 due to a deletion mutation in the CACNA1A gene in a Korean family. Journal of Clinical Neurology. 2006;2(4):268–271.

29. Nachbauer W, Nocker M, Karner E, Stankovic I, Unterberger I, Eigentler A, et al. Episodic ataxia type 2: phenotype characteristics of a novel CACNA1A mutation and review of the literature. Journal of Neurology. 2014 May;261(5):983–91.

30. Zafeiriou DI, Lehmann-Horn F, Vargiami E, Teflioudi E, Ververi A, Jurkat-Rott K. Episodic ataxia type 2 showing ictal hyperhidrosis with hypothermia and interictal chronic diarrhea due to a novel CACNA1A mutation. European Journal of Paediatric Neurology. 2009 Mar;13(2):191–3.

31. Bertholon P, Chabrier S, Riant F, Tournier-Lasserve E, Peyron R. Episodic ataxia type 2: unusual aspects in clinical and genetic presentation. Special emphasis in childhood. Journal of Neurology, Neurosurgery & Psychiatry. 2009 Nov 1;80(11):1289–92.

32. Thiel A, Habedank B, Winhuisen L, Herholz K, Kessler J, Haupt WF, et al. Essential language function of the right hemisphere in brain tumor patients. Annals of neurology. 2005;57(1):128–131.

33. Choi J-H, Seo J-D, Choi YR, Kim M-J, Shin J-H, Kim JS, et al. Exercise-induced downbeat nystagmus in a Korean family with a nonsense mutation in CACNA1A. Neurological Sciences. 2015 Aug;36(8):1393–6.

34. Ophoff RA, Terwindt GM, Vergouwe MN, Van Eijk R, Oefner PJ, Hoffman SM, et al. Familial hemiplegic migraine and episodic ataxia type-2 are caused by mutations in the Ca 2+ channel gene CACNL1A4. Cell. 1996;87(3):543–552.

35. Melzer N, Classen J, Reiners K, Buttmann M. Fluctuating neuromuscular transmission defects and inverse acetazolamide response in episodic ataxia type 2 associated with the novel CaV2.1 single amino acid substitution R2090Q. Journal of the Neurological Sciences. 2010 Sep;296(1–2):104–6.

36. Spacey SD, Hildebrand ME, Materek LA, Bird TD, Snutch TP. Functional implications of a novel EA2 mutation in the P/Q-type calcium channel. Annals of Neurology. 2004 Aug;56(2):213–20.

37. Rajakulendran S, Graves TD, Labrum RW, Kotzadimitriou D, Eunson L, Davis MB, et al. Genetic and functional characterisation of the P/Q calcium channel in episodic ataxia with epilepsy: Variation of CACNA1A in episodic ataxia and epilepsy. The Journal of Physiology. 2010 Jun 1;588(11):1905–13.

38. Denier C, Ducros A, Vahedi K, Joutel A, Thierry P, Ritz A, et al. High prevalence of CACNA1A truncations and broader clinical spectrum in episodic ataxia type 2. Neurology. 1999;52(9):1816–1816.

39. Hu Y, Jiang H, Wang Q, Xie Z, Pan S. Identification of a novel nonsense mutation p. Tyr1957Ter of CACNA1A in a Chinese family with episodic ataxia 2. PloS one. 2013;8(2):e56362.

40. Riant F, Lescoat C, Vahedi K, Kaphan E, Toutain A, Soisson T, et al. Identification of CACNA1A large deletions in four patients with episodic ataxia. neurogenetics. 2010 Feb;11(1):101–6.

41. Mantuano E, Romano S, Veneziano L, Gellera C, Castellotti B, Caimi S, et al. Identification of novel and recurrent CACNA1A gene mutations in fifteen patients with episodic ataxia type 2. Journal of the Neurological Sciences. 2010 Apr;291(1–2):30–6.

42. Scoggan KA, Chandra T, Nelson R, Hahn AF, Bulman DE. Identification of two novel mutations in the CACNA1A gene responsible for episodic ataxia type 2. J Med Genet. 2001 Apr;38(4):249–53.

43. Rucker JC, Jen J, Stahl JS, Natesan N, Baloh RBW, Leigh RJ. Internuclear Ophthalmoparesis in Episodic Ataxia Type 2. Annals of the New York Academy of Sciences. 2005 Apr;1039(1):571–4.

44. Riant F, Mourtada R, Saugier-Veber P, Tournier-Lasserve E. Large CACNA1A deletion in a family with episodic ataxia type 2. Archives of neurology. 2008;65(6):817–820.

45. Wan, Jijun, et al. "Large genomic deletions in CACNA1A cause episodic ataxia type 2." Frontiers in neurology 2 (2011): 51.

46. Labrum RW, Rajakulendran S, Graves TD, Eunson LH, Bevan R, Sweeney MG, et al. Large scale calcium channel gene rearrangements in episodic ataxia and hemiplegic migraine: implications for diagnostic testing. Journal of Medical Genetics. 2009 Nov 1;46(11):786–91.

47. Cuenca-Leon E, Banchs I, Serra SA, Latorre P, Fernandez-Castillo N, Corominas R, et al. Late-onset episodic ataxia type 2 associated with a novel loss-of-function mutation in the CACNA1A gene. Journal of the Neurological Sciences. 2009 May;280(1–2):10–4.

48. Imbrici P, Eunson LH, Graves TD, Bhatia KP, Wadia NH, Kullmann DM, et al. Late-onset episodic ataxia type 2 due to an in-frame insertion in CACNA1A. Neurology. 2005 Sep 27;65(6):944–6.

49. Jen J, Wan J, Graves M, Yu H, Mock AF, Coulin CJ, et al. Loss-of-function EA2 mutations are associated with impaired neuromuscular transmission. Neurology. 2001 Nov 27;57(10):1843–8.

50. Denier C, Ducros A, Durr A, Eymard B, Chassande B, Tournier-Lasserve E. Missense CACNA1A mutation causing episodic ataxia type 2. Arch Neurol. 2001 Feb;58(2):292–5.

51. Eunson LH, Graves TD, Hanna MG. New calcium channel mutations predict aberrant RNA splicing in episodic ataxia. Neurology. 2005;65(2):308–310.

52. Nikaido K, Tachi N, Ohya K, Wada T, Tsutsumi H. New mutation of CACNA1A gene in episodic ataxia type 2: Letter to the editor. Pediatrics International. 2011 Jun;53(3):415–6.

53. Maksemous, Neven, et al. "Next‐generation sequencing identifies novel CACNA 1A gene mutations in episodic ataxia type 2." Molecular genetics & genomic medicine 4.2 (2016): 211-222.

54. Wan J, Carr JR, Baloh RW, Jen JC. Nonconsensus intronic mutations cause episodic ataxia. Ann Neurol. 2005 Jan;57(1):131–5.

55. Subramony SH, Schott K, Raike RS, Callahan J, Langford LR, Christova PS, et al. Novel CACNA1A mutation causes febrile episodic ataxia with interictal cerebellar deficits. Annals of neurology. 2003;54(6):725–731.

56. Kipfer S, Jung S, Lemke JR, Kipfer-Kauer A, Howell JP, Kaelin-Lang A, et al. Novel CACNA1A mutation (s) associated with slow saccade velocities. Journal of neurology. 2013;260(12):3010–3014.

57. Kinder S, Ossig C, Wienecke M, Beyer A, von der Hagen M, Storch A, et al. Novel frameshift mutation in the CACNA1A gene causing a mixed phenotype of episodic ataxia and familiar hemiplegic migraine. European Journal of Paediatric Neurology. 2015 Jan;19(1):72–4.

58. Petrovicova, Andrea, et al. "Novel missense variant of CACNA1A gene in a Slovak family with episodic ataxia type 2." Biomedical Papers 161.1 (2017): 107-110.

59. Miettinen PJ, Vesa J, Orpana A, Palotie A, F?rkkil? M, Wessman M, et al. Novel splice site CACNA1A mutation causing episodic ataxia type 2. neurogenetics. 2004 Feb 1;5(1):69–73.

60. Choi K-D, Yook J-W, Kim M-J, Kim H-S, Park Y-E, Kim JS, et al. Possible anticipation associated with a novel splice site mutation in episodic ataxia type 2. Neurological Sciences. 2013;34(9):1629–1632.

61. Graves TD, Imbrici P, Kors EE, Terwindt GM, Eunson LH, Frants RR, et al. Premature stop codons in a facilitating EF-hand splice variant of CaV2.1 cause episodic ataxia type 2. Neurobiology of Disease. 2008 Oct;32(1):10–5.

62. Yue Q, Jen JC, Nelson SF, Baloh RW. Progressive ataxia due to a missense mutation in a calcium-channel gene. The American Journal of Human Genetics. 1997;61(5):1078–1087.

63. Marti S, Baloh RW, Jen JC, Straumann D, Jung HH. Progressive Cerebellar Ataxia with Variable Episodic Symptoms &ndash; Phenotypic Diversity of R1668W CACNA1A Mutation. European Neurology. 2008;60(1):16–20.

64. Kim H-J, Kim J-S, Choi J-H, Shin J-H, Choi K-D, Zee DS. Rebound Upbeat Nystagmus After Lateral Gaze in Episodic Ataxia Type 2. The Cerebellum. 2014 Jun;13(3):411–3.

65. Strupp M, Kalla R, Dichgans M, Freilinger T, Glasauer S, Brandt T. Treatment of episodic ataxia type 2 with the potassium channel blocker 4-aminopyridine. Neurology. 2004;62(9):1623–1625.

66. Spacey SD, Materek LA, Szczygielski BI, Bird TD. Two novel CACNA1A gene mutations associated with episodic ataxia type 2 and interictal dystonia. Archives of neurology. 2005;62(2):314–316.

67. Isaacs, David Alan, et al. "Case report of novel CACNA1A gene mutation causing episodic ataxia type 2." SAGE open medical case reports 5 (2017): 2050313X17706044.

68. Sintas, Cèlia, et al. "Mutation spectrum in the CACNA1A gene in 49 patients with episodic ataxia." Scientific reports 7.1 (2017): 1-9.

69. Lee, H., et al. "Effectiveness of levetiracetam in an acetazolamide-unresponsive patient with episodic ataxia type 2 by a novel CACNA1A nonsense mutation." European journal of neurology 24.7 (2017): e43-e44.

70. Shimmura, Mitsunori, et al. "Slowed abduction during smooth pursuit eye movement in episodic ataxia type 2 with a novel CACNA1A mutation." Journal of the neurological sciences 381 (2017): 4-6.

71. Choi, Kwang-Dong, et al. "Genetic variants associated with episodic ataxia in Korea." Scientific reports 7.1 (2017): 1-11.

72. Balck, Alexander, et al. "A novel frameshift CACNA1A mutation causing episodic ataxia type 2." The Cerebellum 17.4 (2018): 504-506.

73. Grieco, G. S., et al. "New CACNA1A deletions are associated to migraine phenotypes." The journal of headache and pain 19.1 (2018): 1-6.

74. Lance, Sean, Stuart Mossman, and Gemma Poke. "A Novel CACNA1A Nonsense Variant [c. 4054C> T (p. Arg1352⁎)] Causing Episodic Ataxia Type 2." Case reports in neurological medicine 2018 (2018).

75. Park, Donghwi, et al. "A novel CACNA1A mutation associated with episodic ataxia 2 presenting with periodic paralysis." Acta Neurologica Belgica 118.1 (2018): 137-139.

76. Angelini, Chloé, et al. "Major intra-familial phenotypic heterogeneity and incomplete penetrance due to a CACNA1A pathogenic variant." European journal of medical genetics 62.6 (2019): 103530.

77. Ahuja, Abhimanyu S., Todd D. Rozen, and Paldeep S. Atwal. "A sleep modulated Channelopathy: a novel CACNA1A pathogenic variant identified in episodic Ataxia type 2 and a potential link to sleep alleviated migraine." BMC neurology 19.1 (2019): 1-3.

78. Algahtani, Hussein, et al. "A novel mutation in CACNA1A gene in a Saudi female with episodic ataxia type 2 with no response to acetazolamide or 4-aminopyridine." Intractable & rare diseases research 8.1 (2019): 67-71.

79. Orsucci, Daniele, et al. "Therapy of episodic ataxias: Case report and review of the literature." Drugs in context 8 (2019).

80. Nardello, Rosaria, et al. "Two distinct phenotypes, hemiplegic migraine and episodic Ataxia type 2, caused by a novel common CACNA1A variant." BMC neurology 20.1 (2020): 1-7.

81. Humbertclaude, Véronique, et al. "Cognitive impairment in children with CACNA 1A mutations." Developmental Medicine & Child Neurology 62.3 (2020): 330-337.

82. Zhang, Linxia, et al. "CACNA1A Gene Variants in Eight Chinese Patients With a Wide Range of Phenotypes." Frontiers in Pediatrics 8 (2020).

83. Batum, Melike, et al. "Coincidental occurance of episodic ataxia and multiple sclerosis: a case report and review of the literature." International Journal of Neuroscience (2020): 1-6.

84. Kim, Minkyeong, et al. "Clarification of undiagnosed ataxia using whole-exome sequencing with clinical implications." Parkinsonism & Related Disorders 80 (2020): 58-64.

85. Penkava, Josef, et al. "A novel pathogenic CACNA1A variant causing episodic ataxia type 2 (EA2) spectrum phenotype in four family members and a novel combined therapy." Journal of neurology 267.1 (2020): 181-184.

86. Wu, H. J., et al. "Differentiating episodic ataxia type 2 from migraine: a case report." Hong Kong Medical Journal= Xianggang yi xue za zhi 26.6 (2020): 526-527.

87. Idiculla, Pretty Sara, and Junaid Habib Siddiqui. "A case of novel CACNA1A mutation causing type 2 episodic ataxia." Neurological Sciences 42.6 (2021): 2577-2578.

88. Indelicato, Elisabetta, et al. "The electrophysiological footprint of CACNA1A disorders." Journal of Neurology (2021): 1-13.

89. Verriello, Lorenzo, et al. "Epilepsy and episodic ataxia type 2: family study and review of the literature." Journal of Neurology (2021): 1-7.

90. Verriello, Lorenzo, et al. "Case report and ten-year follow-up of episodic ataxia type 2 due to a novel variant in CACNA1A." Eneurologicalsci 23 (2021).

91. Na, Seunghee, and Taewon Kim. "Efficacy of levetiracetam in patients with episodic ataxia type 2 caused by CACNA1A mutation: three case reports." Neurological Sciences (2021): 1-3.

92. Pal, Deb K., and Ruth E. Williams. "Response to pyridoxine in CACNA1A epilepsy-ataxia does not imply a causal effect." Seizure 91 (2021): 196-197.

93. Le Roux, Marie, et al. "CACNA1A-associated epilepsy: Electroclinical findings and treatment response on seizures in 18 patients." European Journal of Paediatric Neurology (2021).

94. Hommersom MP, van Prooije TH, Pennings M, Schouten MI, van Bokhoven H, Kamsteeg EJ, van de Warrenburg BPC. The complexities of CACNA1A in clinical neurogenetics. J Neurol. 2021 Nov 22.

95. Niu, X., Yang, Y., Chen, Y., Cheng, M., Liu, M., Ding, C., Tian, X., Yang, Z., Jiang, Y., & Zhang, Y. (2022). Genotype–phenotype correlation of CACNA1A variants in children with epilepsy. Developmental Medicine and Child Neurology, 64(1), 105–111.

96. Brunklaus A. Advances in genotype-phenotype associations for CACNA1A-related epilepsies. Eur J Paediatr Neurol. 2021 Jul;33:A2. doi: 10.1016/j.ejpn.2021.07.001. Epub 2021 Jul 3.

97. Indelicato E, Boesch S. From Genotype to Phenotype: Expanding the Clinical Spectrum of CACNA1A Variants in the Era of Next Generation Sequencing. Front Neurol. 2021 Mar 2;12:639994.

98. Jaudon, F., Baldassari, S., Musante, I., Thalhammer, A., Zara, F., & Cingolani, L. A. (2020). Targeting alternative splicing as a potential therapy for episodic ataxia type 2. Biomedicines, 8(9), 1–23.

99. Stendel, C., D’Adamo, M. C., Wiessner, M., Dusl, M., Cenciarini, M., Belia, S., Nematian-Ardestani, E., Bauer, P., Senderek, J., Klopstock, T., & Pessia, M. (2020). Association of a novel splice site mutation in P/Q-type calcium channels with childhood epilepsy and late-onset slowly progressive non-episodic cerebellar ataxia. International Journal of Molecular Sciences, 21(11)

100. Giunti, P., Mantuano, E., & Frontali, M. (2020). Episodic ataxias: Faux or real? International Journal of Molecular Sciences, 21(18), 1–16.

101. Kipfer, S., & Strupp, M. (2014). The Clinical Spectrum of Autosomal-Dominant Episodic Ataxias. Movement Disorders Clinical Practice, 1(4), 285–290.

102. Travaglini, L., Nardella, M., Bellacchio, E., D’Amico, A., Capuano, A., Frusciante, R., Di Capua, M., Cusmai, R., Barresi, S., Morlino, S., Fernández-Fernández, J. M., Trivisano, M., Specchio, N., Valeriani, M., Vigevano, F., Bertini, E., & Zanni, G. (2017). Missense mutations of CACNA1A are a frequent cause of autosomal dominant nonprogressive congenital ataxia. European Journal of Paediatric Neurology, 21(3), 450–456.

103. Kurosaki, T., & Maquat, L. E. (2016). Nonsense-mediated mRNA decay in humans at a glance. Journal of Cell Science, 129(3), 461–467.

104. Sintas, C., Carreño, O., Fernàndez-Castillo, N., Corominas, R., Vila-Pueyo, M., Toma, C., Cuenca-León, E., Barroeta, I., Roig, C., Volpini, V., MacAya, A., & Cormand, B. (2017). Mutation Spectrum in the CACNA1A Gene in 49 Patients with Episodic Ataxia. Scientific Reports, 7(1), 1–9.

List of recovered articles – Validation Dataset

## EA 1 - *KCNA1*

1. Sun WB, Fu JX, Chen YL, Li HF, Wu ZY, Chen DF. Both gain- and loss-of-function variants of KCNA1 are associated with paroxysmal kinesigenic dyskinesia. J Genet Genomics. 2024 Aug;51(8):801-810. doi: 10.1016/j.jgg.2024.03.013. Epub 2024 Apr 2. PMID: 38570113.

## EA 2 – *CACNA1A*

1. Lipman AR, Fan X, Shen Y, Chung WK. Clinical and genetic characterization of CACNA1A-related disease. Clin Genet. 2022 Oct;102(4):288-295. doi: 10.1111/cge.14180. Epub 2022 Jun 26. PMID: 35722745; PMCID: PMC9458680.
2. González-Mingot C, López-Ortega R, Brieva-Ruiz L. The efficacy of combining topiramate and 4-aminopyridine to reduce relapses and interictal progression in two cases of episodic ataxia type 2. Neurol Sci. 2022 Aug;43(8):5099-5101. doi: 10.1007/s10072-022-06144-2. Epub 2022 May 20. PMID: 35595874.
3. Argenziano G, Cavallieri F, Monfrini E, Gessani A, Russo M, Rizzi R, Fioravanti V, Grisanti S, Toschi G, Napoli M, Pascarella R, Budriesi C, Di Fonzo A, Zucco R, Valzania F. Deconstructing speech alterations in episodic ataxia type 2: Perceptual-acoustic analysis in a case due to CACNA1A gene mutation. Parkinsonism Relat Disord. 2023 Mar;108:105311. doi: 10.1016/j.parkreldis.2023.105311. Epub 2023 Feb 13. PMID: 36805178.
4. Lu X, Xie X. EA2 and temporal lobe epilepsy associated with a novel variant in CACNA1A. Seizure. 2023 May;108:10-12. doi: 10.1016/j.seizure.2023.03.019. Epub 2023 Apr 6. PMID: 37059034.
5. Alcalá-Torres J, Pérez-de la Fuente R, Cárdenas-Del Carre A, Arteche-López A, Posada-Rodríguez IJ. Ataxia episódica tipo 2: estudio clínico, genético y radiológico de 10 pacientes [Episodic ataxia type 2: a clinical, genetic and radiological study of 10 patients]. Rev Neurol. 2023 May 16;76(10):321-325. Spanish. doi: 10.33588/rn.7610.2023117. PMID: 37165528; PMCID: PMC10478108.
6. Riant F, Burglen L, Corpechot M, Robert J, Durr A, Solé G, Petit F, Freihuber C, De Marco O, Sarret C, Castelnovo G, Devillard F, Afenjar A, Héron B, Lasserve ET. Characterization of novel CACNA1A splice variants by RNA-sequencing in patients with episodic or congenital ataxia. Clin Genet. 2023 Sep;104(3):365-370. doi: 10.1111/cge.14358. Epub 2023 May 13. PMID: 37177896.
7. Martínez-Rubio D, Hinarejos I, Argente-Escrig H, Marco-Marín C, Lozano MA, Gorría-Redondo N, Lupo V, Martí-Carrera I, Miranda C, Vázquez-López M, García-Pérez A, Marco-Hernández AV, Tomás-Vila M, Aguilera-Albesa S, Espinós C. Genetic Heterogeneity Underlying Phenotypes with Early-Onset Cerebellar Atrophy. Int J Mol Sci. 2023 Nov 16;24(22):16400. doi: 10.3390/ijms242216400. PMID: 38003592; PMCID: PMC10671053.
8. Mascherpa M, Fichera A, Orabona R, Recupero D, Borroni B, Odicino FE, Prefumo F. Inherited episodic ataxia type 2 in pregnancy: A case report. Int J Gynaecol Obstet. 2024 Apr;165(1):387-389. doi: 10.1002/ijgo.15292. Epub 2023 Dec 7. PMID: 38059694.
9. Miura S, Watanabe E, Senzaki K, Hiruki S, Matsumoto S, Morikawa T, Uchiyama Y, Kurata S, Ochi M, Ohyagi Y, Shibata H. Episodic ataxia type 2 with a novel missense variant (Leu602Arg) in CACNA1A. Hum Genome Var. 2024 Jan 15;11(1):3. doi: 10.1038/s41439-023-00261-w. PMID: 38221525; PMCID: PMC10788331.
10. Kim S, Choi JY, Kim JS, Kim HJ. Torsional saccadic palsy in episodic ataxia type 2. J Neurol. 2024 Oct;271(10):7039-7041. doi: 10.1007/s00415-024-12636-2. Epub 2024 Sep 6. PMID: 39242370.
11. Kim S, Kim JS, Lee SH, Kim JM, Na S, Choi JH, Kim HJ. Intellectual Disability in Episodic Ataxia Type 2: Beyond Paroxysmal Vertigo and Ataxia. J Clin Neurol. 2024 Nov;20(6):563-570. doi: 10.3988/jcn.2024.0274. PMID: 39505308; PMCID: PMC11543395.
12. Verriello L, Pez S, Pauletto G, Valente M. Efficacy and safety of 4-aminopyridine in episodic ataxia type 2: a case series. J Neurol. 2025 Feb 15;272(3):205. doi: 10.1007/s00415-025-12941-4. PMID: 39954114.
13. Bozkaya-Yilmaz S, Olgac-Dundar N, Aliyeva N, Ersen A, Gencpinar P, Gungor M, Hiz AS, Yis U, Sarikaya-Uzan G, Sarigecili E, Kirik S, Erol I, Besen S, Kayilioglu H, Haspolat S, Kipoglu O, Ekici A, Turay S, Tosun A, Ayanoglu M, Danis A, Hancı F, Kutbay YB, Ozyilmaz B, Kara B. Phenotypic variability in cases with CACNA1A mutation. Eur J Pediatr. 2025 Mar 20;184(4):261. doi: 10.1007/s00431-025-06062-3. PMID: 40111503; PMCID: PMC11926052.
14. Shimazaki H. Effects of Levetiracetam on Episodic Ataxia Type 2 and Spinocerebellar Ataxia Type 6 with Episodic Ataxic Symptoms: A Case Series. Genes (Basel). 2025 Mar 13;16(3):335. doi: 10.3390/genes16030335. PMID: 40149486; PMCID: PMC11942281.
