# Supplementary material for: Diagnostic approach to episodic ataxia types 1 and 2: a proposed algorithm for limited resource-settings
Source: Front Neurol. 2026 Apr 21;17:1735246. doi: 10.3389/fneur.2026.1735246 (PMC13141855; doi:10.3389/fneur.2026.1735246)
Supplement: Supplementary file 2 [file Supplementary_file_2.docx]

**Supplementary Material S2 – Variant Frequency, Distribution and Functional Effect**

*KCNA1*

The most frequent variants were p.V174F (16/94 or 17%), p.F250del (9/94 or 9.6%), p.I407M (7/94 or 7.4%) and p.F414S (7/94 or 7.4%). The majority of individuals had missense variants (84/94, 89.4%). Variant effect was unknown in 42.5% of individuals (40/94). When disease mechanism was known, it was most commonly LoF (53/94 individuals or 56.4%, comprising 48.9% missense, 6.4% dominant negative and 1.1% haploinsufficiency). Only one variant had a known GoF effect (p.A261T).

Variant frequency : ***KCNA1***

|  | | | | |
| --- | --- | --- | --- | --- |
| c.DNA | Frequency | Percent | Valid Percent | Cumulative Percent |
| c.520G>T | 16 | 17,0 | 17,0 | 17,0 |
| c.748_750delTTC | 9 | 9,6 | 9,6 | 26,6 |
| c.1221T>G | 7 | 7,4 | 7,4 | 34,0 |
| c.1241T>C | 7 | 7,4 | 7,4 | 41,5 |
| c.677C>G | 6 | 6,4 | 6,4 | 47,9 |
| c.746T>G | 6 | 6,4 | 6,4 | 54,3 |
| c.941T>C | 6 | 6,4 | 6,4 | 60,6 |
| c.975G>C | 6 | 6,4 | 6,4 | 67,0 |
| c.1241T>G | 4 | 4,3 | 4,3 | 71,3 |
| c.919C>T | 4 | 4,3 | 4,3 | 75,5 |
| c.555C>G | 3 | 3,2 | 3,2 | 78,7 |
| c.724G>C | 3 | 3,2 | 3,2 | 81,9 |
| c.973G>C | 3 | 3,2 | 3,2 | 85,1 |
| c.500G>T | 2 | 2,1 | 2,1 | 87,2 |
| c.1025G>T | 1 | 1,1 | 1,1 | 88,3 |
| c.1210G>A | 1 | 1,1 | 1,1 | 89,4 |
| c.1249C>T | 1 | 1,1 | 1,1 | 90,4 |
| c.521T>C | 1 | 1,1 | 1,1 | 91,5 |
| c.677C>T | 1 | 1,1 | 1,1 | 92,6 |
| c.781G>A | 1 | 1,1 | 1,1 | 93,6 |
| c.785T>C | 1 | 1,1 | 1,1 | 94,7 |
| c.786C>G | 1 | 1,1 | 1,1 | 95,7 |
| c.847G>A | 1 | 1,1 | 1,1 | 96,8 |
| c.895G>A | 1 | 1,1 | 1,1 | 97,9 |
| c.913C>T | 1 | 1,1 | 1,1 | 98,9 |
| c.932G>A | 1 | 1,1 | 1,1 | 100,0 |
| Total | 94 | 100,0 | 100,0 |  |

Variant functional effect: ***KCNA1***

|  |  |  |  |  |  |  |
| --- | --- | --- | --- | --- | --- | --- |
|  | | Gain of function vs Loss of Funcion | | | | Total |
|  |  | LoF | GoF | Unkwown | Dominant negative |  |
| VARIANT TYPE COD | Missense | 46 | 1 | 31 | 6 | 84 |
|  | Nonsense | 1 | 0 | 0 | 0 | 1 |
|  | In frame deletion | 0 | 0 | 9 | 0 | 9 |
| Total | | 47 | 1 | 40 | 6 | 94 |

**CACNA1A**

In the core dataset, there were 133 distinct variants in *CACNA1A*, spread throughout the gene without a clear mutational hotspot. The most frequent variants were p.R1546* (5.1%), c.5629-2A>G (4.7%), c.978+1G>A (4%) and c.3092+2T>C (3.3%). All other variants had a frequency less than 3%. The most frequent types of variants were missense (26.8%), nonsense (24.6%) and splicing (21.7%) Disease mechanism was unknown in 83/276 individuals (30%, mostly comprised of missense variants). When disease mechanism was known, LoF variants predominated (191/276 or 69.2%, comprising truncating/haploinsufficiency variants in 62.3%, missense 5%, dominant negative 1.1% and in-frame insertions/deletions in 0.8%). Only one variant was identified to have a GoF effect (p.T501M; affecting two individuals or 0.8%)

Variant Frequency : ***CACNA1A***

|  | Frequency | Percent | Valid Percent | Cumulative Percent |
| --- | --- | --- | --- | --- |
| c.4636C>T | 14 | 5,1 | 5,1 | 5,1 |
| c.5629-2A>G | 13 | 4,7 | 4,7 | 9,8 |
| c.978+1G>A | 11 | 4,0 | 4,0 | 13,8 |
| c.3092+2T>C | 9 | 3,3 | 3,3 | 17,0 |
| c.2042_2043del | 6 | 2,2 | 2,2 | 19,2 |
| c.4343G>A | 6 | 2,2 | 2,2 | 21,4 |
| c.4392-1G>C | 6 | 2,2 | 2,2 | 23,6 |
| c.4461_4466del | 6 | 2,2 | 2,2 | 25,7 |
| c.2206C>T | 5 | 1,8 | 1,8 | 27,5 |
| c.3832C>T | 5 | 1,8 | 1,8 | 29,3 |
| c.835C>T | 5 | 1,8 | 1,8 | 31,2 |
| c.860G>A | 5 | 1,8 | 1,8 | 33,0 |
| c.904G>A | 5 | 1,8 | 1,8 | 34,8 |
| c.1597G>A | 4 | 1,4 | 1,4 | 36,2 |
| c.3460C>T | 4 | 1,4 | 1,4 | 37,7 |
| c.4845del | 4 | 1,4 | 1,4 | 39,1 |
| c.766T>C | 4 | 1,4 | 1,4 | 40,6 |
| c.1747C>T | 3 | 1,1 | 1,1 | 41,7 |
| c.3695+1G>A | 3 | 1,1 | 1,1 | 42,8 |
| c.3846C>G | 3 | 1,1 | 1,1 | 43,8 |
| c.3992+1G>A | 3 | 1,1 | 1,1 | 44,9 |
| c.4107T>A | 3 | 1,1 | 1,1 | 46,0 |
| c.439G>A | 3 | 1,1 | 1,1 | 47,1 |
| c.4953+1374_*3804del | 3 | 1,1 | 1,1 | 48,2 |
| c.5137–10_5137–2del | 3 | 1,1 | 1,1 | 49,3 |
| c.5267A>G | 3 | 1,1 | 1,1 | 50,4 |
| c.5871C>A | 3 | 1,1 | 1,1 | 51,4 |
| c.889G>A | 3 | 1,1 | 1,1 | 52,5 |
| c.1063dup | 2 | 0,7 | 0,7 | 53,3 |
| c.1162G>A | 2 | 0,7 | 0,7 | 54,0 |
| c.1363C>T | 2 | 0,7 | 0,7 | 54,7 |
| c.1502C>T | 2 | 0,7 | 0,7 | 55,4 |
| c.1618G>C | 2 | 0,7 | 0,7 | 56,2 |
| c.1872_1875del | 2 | 0,7 | 0,7 | 56,9 |
| c.1913G>A | 2 | 0,7 | 0,7 | 57,6 |
| c.2259_2260insCG | 2 | 0,7 | 0,7 | 58,3 |
| c.4036C>T | 2 | 0,7 | 0,7 | 59,1 |
| c.4208T>G | 2 | 0,7 | 0,7 | 59,8 |
| c.4469T>C | 2 | 0,7 | 0,7 | 60,5 |
| c.4681C>T | 2 | 0,7 | 0,7 | 61,2 |
| c.4953+1G>A | 2 | 0,7 | 0,7 | 62,0 |
| c.4990C>T | 2 | 0,7 | 0,7 | 62,7 |
| c.5035C>T | 2 | 0,7 | 0,7 | 63,4 |
| c.5251C>T | 2 | 0,7 | 0,7 | 64,1 |
| c.5455C>T | 2 | 0,7 | 0,7 | 64,9 |
| c.5569C>T | 2 | 0,7 | 0,7 | 65,6 |
| c.5629-1151_5943+452del | 2 | 0,7 | 0,7 | 66,3 |
| c.5735-3415_*9966del | 2 | 0,7 | 0,7 | 67,0 |
| c.743A>G | 2 | 0,7 | 0,7 | 67,8 |
| c.877G>A | 2 | 0,7 | 0,7 | 68,5 |
| c2070_2071delinsGGAG | 2 | 0,7 | 0,7 | 69,2 |
| del: exon 4 | 2 | 0,7 | 0,7 | 69,9 |
| del: exons 39–47 | 2 | 0,7 | 0,7 | 70,7 |
| deletion exons 12–15 | 2 | 0,7 | 0,7 | 71,4 |
| c.4999C>T | 1 | 0,4 | 0,4 | 71,7 |
| <7474 bp incl ex. 27 | 1 | 0,4 | 0,4 | 72,1 |
| c.1159C>G | 1 | 0,4 | 0,4 | 72,5 |
| c.1165C>T | 1 | 0,4 | 0,4 | 72,8 |
| c.1231G>T | 1 | 0,4 | 0,4 | 73,2 |
| c.1364G>A | 1 | 0,4 | 0,4 | 73,6 |
| c.1558+1G>A | 1 | 0,4 | 0,4 | 73,9 |
| c.1642del | 1 | 0,4 | 0,4 | 74,3 |
| c.165dup | 1 | 0,4 | 0,4 | 74,6 |
| c.1672-1_1675delGGTTA | 1 | 0,4 | 0,4 | 75,0 |
| c.1748G>A | 1 | 0,4 | 0,4 | 75,4 |
| c.1799_1800delTC | 1 | 0,4 | 0,4 | 75,7 |
| c.1862T>G | 1 | 0,4 | 0,4 | 76,1 |
| c.1997C>T | 1 | 0,4 | 0,4 | 76,4 |
| c.202C>T | 1 | 0,4 | 0,4 | 76,8 |
| c.2030G>A | 1 | 0,4 | 0,4 | 77,2 |
| c.2042del | 1 | 0,4 | 0,4 | 77,5 |
| c.2393T>C | 1 | 0,4 | 0,4 | 77,9 |
| c.2464dup | 1 | 0,4 | 0,4 | 78,3 |
| c.2496dup | 1 | 0,4 | 0,4 | 78,6 |
| c.2690C>G | 1 | 0,4 | 0,4 | 79,0 |
| c.2691dup | 1 | 0,4 | 0,4 | 79,3 |
| c.2816del | 1 | 0,4 | 0,4 | 79,7 |
| c.2816dup | 1 | 0,4 | 0,4 | 80,1 |
| c.2847 _2856del | 1 | 0,4 | 0,4 | 80,4 |
| c.2853_2862del10 | 1 | 0,4 | 0,4 | 80,8 |
| c.3000dup | 1 | 0,4 | 0,4 | 81,2 |
| c.3312dup | 1 | 0,4 | 0,4 | 81,5 |
| c.3396_3404dup | 1 | 0,4 | 0,4 | 81,9 |
| c.3414del | 1 | 0,4 | 0,4 | 82,2 |
| c.3414dup | 1 | 0,4 | 0,4 | 82,6 |
| c.3492del | 1 | 0,4 | 0,4 | 83,0 |
| c.3871_3873del | 1 | 0,4 | 0,4 | 83,3 |
| c.3901delC | 1 | 0,4 | 0,4 | 83,7 |
| c.3992+2dup | 1 | 0,4 | 0,4 | 84,1 |
| c.4054C>T | 1 | 0,4 | 0,4 | 84,4 |
| c.4249_4253+2del | 1 | 0,4 | 0,4 | 84,8 |
| c.4250_4253+2del | 1 | 0,4 | 0,4 | 85,1 |
| c.4253+1G>A | 1 | 0,4 | 0,4 | 85,5 |
| c.4253G>A | 1 | 0,4 | 0,4 | 85,9 |
| c.4254–1G>A | 1 | 0,4 | 0,4 | 86,2 |
| c.4391+1G>T | 1 | 0,4 | 0,4 | 86,6 |
| c.464T>C | 1 | 0,4 | 0,4 | 87,0 |
| c.4778_4780del | 1 | 0,4 | 0,4 | 87,3 |
| c.485G>T | 1 | 0,4 | 0,4 | 87,7 |
| c.4889G>A | 1 | 0,4 | 0,4 | 88,0 |
| c.5020C>T | 1 | 0,4 | 0,4 | 88,4 |
| c.504del | 1 | 0,4 | 0,4 | 88,8 |
| c.5070+1G>A | 1 | 0,4 | 0,4 | 89,1 |
| c.5115-5126del | 1 | 0,4 | 0,4 | 89,5 |
| c.5207A>T | 1 | 0,4 | 0,4 | 89,9 |
| c.5253-2259_5403+1135del | 1 | 0,4 | 0,4 | 90,2 |
| c.5266G>A | 1 | 0,4 | 0,4 | 90,6 |
| c.539+39569_*10894del | 1 | 0,4 | 0,4 | 90,9 |
| c.5547T>A | 1 | 0,4 | 0,4 | 91,3 |
| c.5559C>G | 1 | 0,4 | 0,4 | 91,7 |
| c.5605T>C | 1 | 0,4 | 0,4 | 92,0 |
| c.5735-3578_5943+453del | 1 | 0,4 | 0,4 | 92,4 |
| c.5776G>T | 1 | 0,4 | 0,4 | 92,8 |
| c.5941C>T | 1 | 0,4 | 0,4 | 93,1 |
| c.6053+4_6053+7del | 1 | 0,4 | 0,4 | 93,5 |
| c.6190C>T | 1 | 0,4 | 0,4 | 93,8 |
| c.6269G>A | 1 | 0,4 | 0,4 | 94,2 |
| c.6665C>T | 1 | 0,4 | 0,4 | 94,6 |
| c.742T>A | 1 | 0,4 | 0,4 | 94,9 |
| c.749del | 1 | 0,4 | 0,4 | 95,3 |
| c.757C>T | 1 | 0,4 | 0,4 | 95,7 |
| c.758A>G | 1 | 0,4 | 0,4 | 96,0 |
| c.785-2649_979-8957del | 1 | 0,4 | 0,4 | 96,4 |
| c.791del | 1 | 0,4 | 0,4 | 96,7 |
| c.928_931delACTG | 1 | 0,4 | 0,4 | 97,1 |
| c.959G>A | 1 | 0,4 | 0,4 | 97,5 |
| c.979-1G>A | 1 | 0,4 | 0,4 | 97,8 |
| c.979-2453_1348+3157del | 1 | 0,4 | 0,4 | 98,2 |
| Del 4141bp incl ex. 30, ins 22bp | 1 | 0,4 | 0,4 | 98,6 |
| del: exon 27 | 1 | 0,4 | 0,4 | 98,9 |
| del: exon 6 | 1 | 0,4 | 0,4 | 99,3 |
| del: exons 20–38 | 1 | 0,4 | 0,4 | 99,6 |
| deletion exon 47 | 1 | 0,4 | 0,4 | 100,0 |
| Total | 276 | 100,0 | 100,0 |  |

Variant functional effect: ***CACNA1A***

|  | | Gain of function vs Loss of Funcion | | | | Total |
| --- | --- | --- | --- | --- | --- | --- |
|  |  | LoF | GoF | Unkwown | Dominant negative |  |
| VARIANT TYPE COD | Missense | 14 | 2 | 55 | 3 | 74 |
|  | Frameshift | 42 | 0 | 0 | 0 | 42 |
|  | Nonsense | 68 | 0 | 0 | 0 | 68 |
|  | Splicing | 52 | 0 | 8 | 0 | 60 |
|  | In frame deletion | 0 | 0 | 8 | 0 | 8 |
|  | In Frame indel | 1 | 0 | 0 | 0 | 1 |
|  | Large deletion | 10 | 0 | 11 | 0 | 21 |
|  | Complex rearrangement | 0 | 0 | 1 | 0 | 1 |
|  | Inframe insertion | 1 | 0 | 0 | 0 | 1 |
| Total | | 188 | 2 | 83 | 3 | 276 |
