# Supplementary material for: Diagnostic approach to episodic ataxia types 1 and 2: a proposed algorithm for limited resource-settings
Source: Front Neurol. 2026 Apr 21;17:1735246. doi: 10.3389/fneur.2026.1735246 (PMC13141855; doi:10.3389/fneur.2026.1735246)
Supplement: Supplementary file 3 [file Supplementary_file_3.docx]

## Supplementary material S3 - TABLES

**Table – Key clinical and demographic characteristics, Validation cohort**

|  | EA1 | EA2 |
| --- | --- | --- |
| SUBJECTS (N) | 24 | 1 |
| FEMALE SEX (N, %) | 12 (50%) | 1 (100%) |
| AGE OF ONSET (MEDIAN, 25th-75th IQR, [RANGE]) | 10.0, 5.0-13.0, [0.16-55] | 12 |
| SUBJECTS MISSING AGE OF ONSET DATA | 3 (12,5%) | 0 |
| AGE AT PUBLICATION, Y (MEAN ± SD) | 35.58 ± 17.14 | 29 |
| ATTACK DURATION (N, %) |  |  |
| BRIEF (<= 10 MIN) | 0 | 1 (100%) |
| INTERMEDIATE (> 10 and <= 60 min) | 9 (37.5%) | 0 |
| PROLONGED (> 60 min and < 1 day) | 12 (50%) | 0 |
| PROTRACTED (>= 1 day) | 3 (12.5%) | 0 |
| SUBJECTS MISSING ATTACK DURATION DATA | 0 | 0 |
| ATTACK FREQUENCY (N, %) |  |  |
| DAILY | 2 (8.7%) | 1 (100%) |
| WEEKLY | 10 (43.47%) | 0 |
| MONTHLY | 9 (39.13%) | 0 |
| RARE/SPORADIC | 2 (8.7%) | 0 |
| SUBJECTS MISSING ATTACK FREQUENCY DATA | 1 | 0 |

**Table: Frequency of reported symptoms during attack; Core dataset**

| **Symptom** | ***CACNA1A* Absolute freq** | **CACNA1A %** | **KCNA1**  **Absolute freq** | **KCNA1 %** | **p value*** |
| --- | --- | --- | --- | --- | --- |
| Limb ataxia | 25/221 | 11.3 | 46/94 | 48.9 | <0.001 |
| Axial ataxia | 91/221 | 41.2 | 64/94 | 68.1 | <0.001 |
| Vertigo | 139/220 | 63.2 | 22/93 | 23.7 | <0.001 |
| Gastrointestinal symptoms | 98/220 | 44.5 | 8/94 | 8.5 | <0.001 |
| Sensory disturbances | 14/221 | 6.3 | 4/93 | 4.3 | 0.479 |
| Oculomotor and/or visual abnormalities | 47/220 | 21.4 | 32/93 | 34.4 | 0.015 |
| Headache | 55/220 | 25.0 | 4/94 | 4.3 | <0.001 |
| Autonomic symptoms | 8/220 | 3.6 | 4/94 | 4.3 | 0.793 |
| Fatigue | 16/220 | 7.5 | 0/94 | 0 | 0.007 |
| Rigidity/Stiffness | 1/220 | 0.5 | 25/94 | 26.6 | <0.001 |
| Myokymia | 1/220 | 0.5 | 22/94 | 23.4 | <0.001 |

*****Chi Squared

**Table: Frequency of reported symptoms between attacks; Core dataset**

| **Symptom** | **CACNA1A Absolute freq** | **CACNA1A %** | **KCNA1**  **Absolute freq** | **KCNA1 %** | **p value*** |
| --- | --- | --- | --- | --- | --- |
| Any ataxia | 106/246 | 43.1% | 7/80 | 8.8% | <0.001 |
| Abnormal tandem | 20/245 | 8.2% | 2/79 | 2.5% | 0.084 |
| Dysarthria | 18/246 | 7.3% | 0/80 | 0% | 0.013 |
| Dysdiadochokinesia | 11/245 | 4.5% | 1/80 | 1.3% | 0.182 |
| Dysmetria | 11/245 | 4.5% | 0/80 | 0% | 0.054 |
| Progressive ataxia | 19/173 | 11.0% | 0/75 | 0% | 0.003 |
| Peripheral muscular symptoms | 2/246 | 0.8% | 66/80 | 82.5% | <0.001 |
| Headache | 55/220 | 25.0% | 4/94 | 4.3% | < 0.001 |
| Nystagmus | 156/246 | 63.4% | 1/80 | 1.3% | <0.001 |
| Dystonia | 7/246 | 2.8% | 4/80 | 5% | 0.354 |
| Seizures | 20/246 | 8.1% | 6/80 | 7.5% | 0.857 |
| Tremors | 2/246 | 0.8% | 8/80 | 10% | <0.001 |
| Mental retardation | 11/246 | 4.5% | 1/80 | 1.3% | 0.184 |
| Neuromyotonia | 0/246 | 0% | 16/80 | 20% | <0.001 |
| Clinical myokymia | 1/246 | 0.4% | 32/80 | 40% | < 0.001 |
| EMG Myokymia | 0/246 | 0% | 38/79 | 48.1% | <0.001 |
| Cerebellar atrophy on imaging | 38/114 | 33.3% | 4/17 | 23.5% | 0.419 |

*****Chi Squared
