# Supplementary material for: Diagnostic approach to episodic ataxia types 1 and 2: a proposed algorithm for limited resource-settings
Source: Front Neurol. 2026 Apr 21;17:1735246. doi: 10.3389/fneur.2026.1735246 (PMC13141855; doi:10.3389/fneur.2026.1735246)
Supplement: Supplementary file 4 [file Supplementary_file_4.docx]

**Supplemental material S4 – additional figures**

**ROC curve – Age of onset**
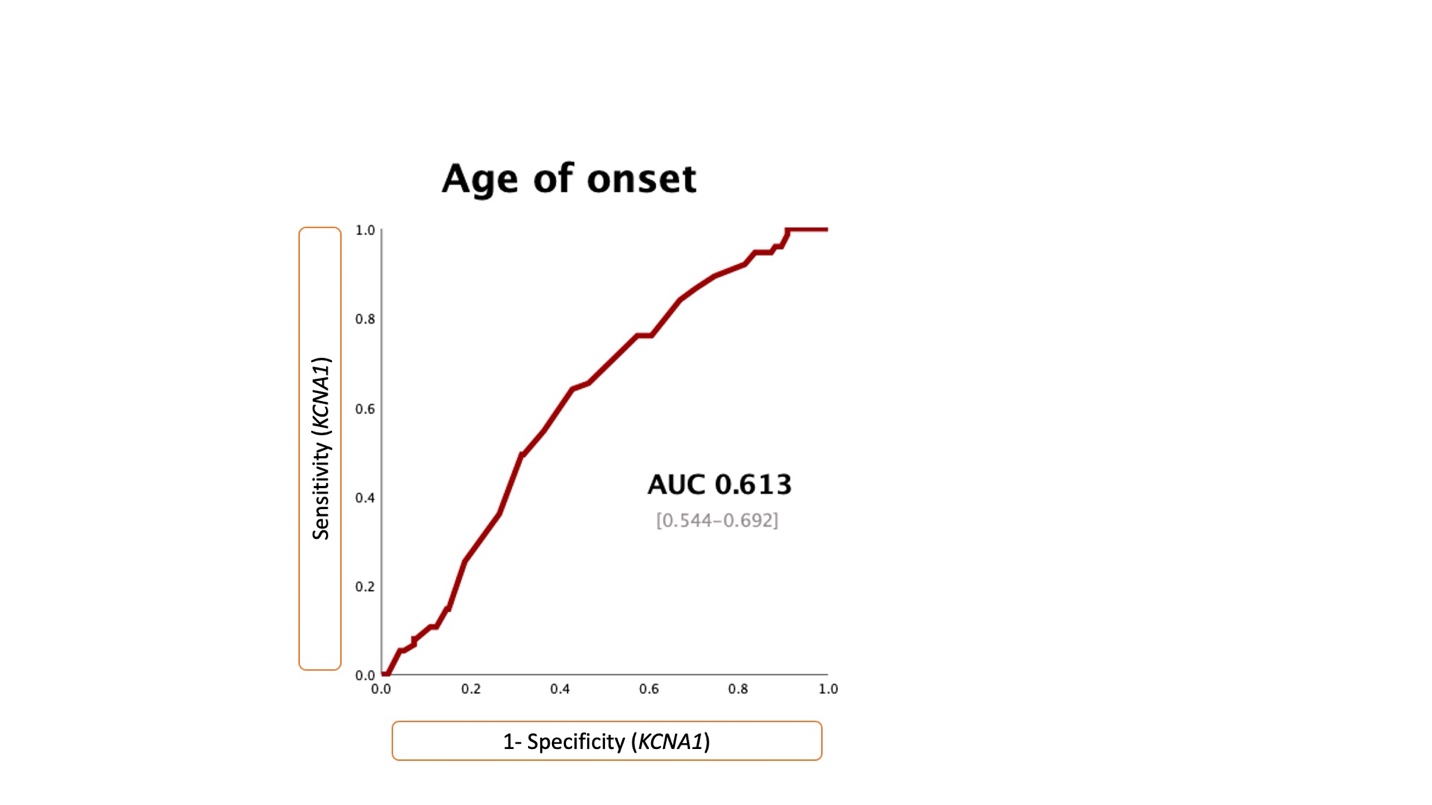


*Key: AUC = area under the curve. Grayed out numbers in brackets represent 95% confidence interval.*

**ROC curve – Attack frequency**
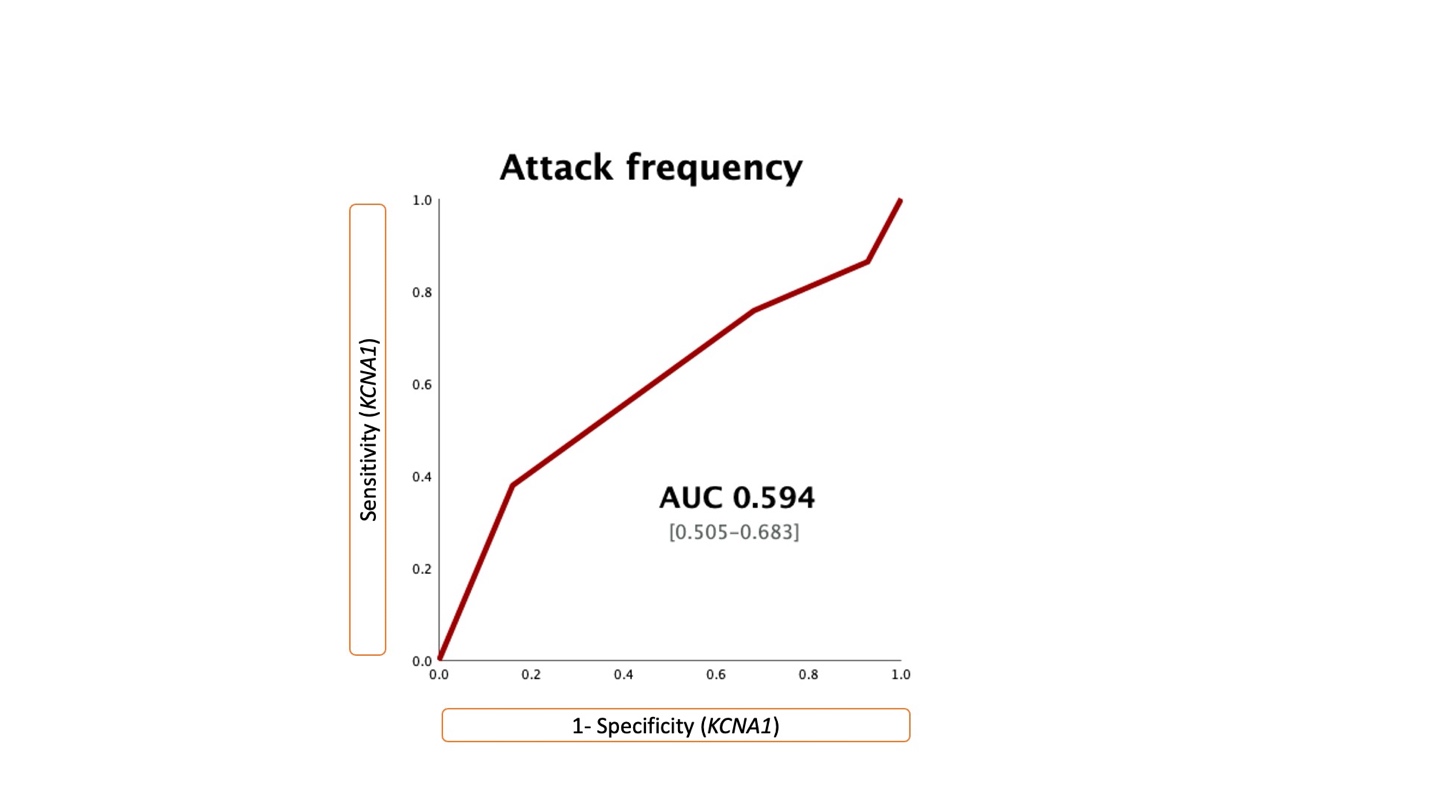


*Key: AUC = area under the curve. Grayed out numbers in brackets represent 95% confidence interval.*

**ROC curve – Acetazolamide response**


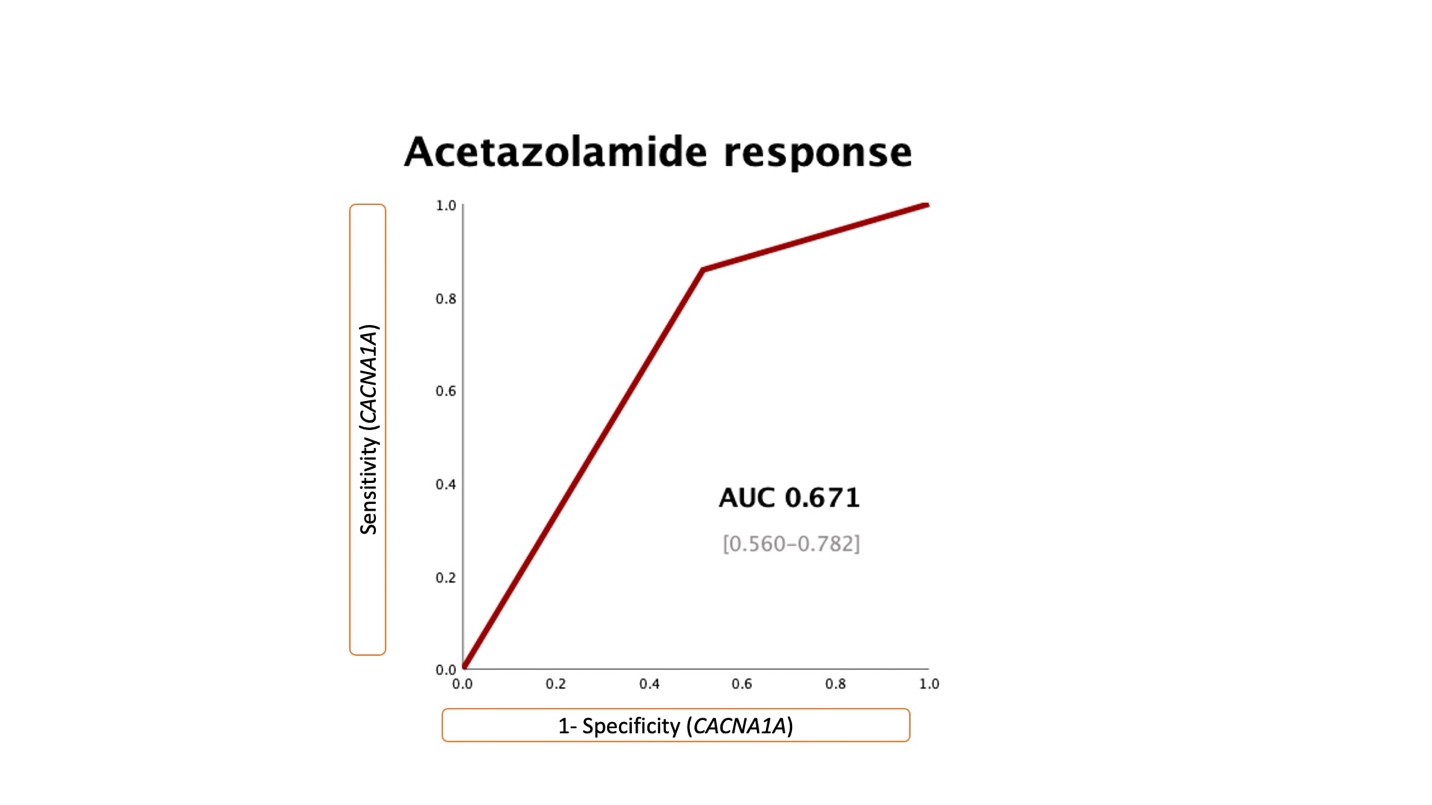


*Key: AUC = area under the curve. Grayed out numbers in brackets represent 95% confidence interval.*
