# Supplementary figures and images for: Diagnostic approach to episodic ataxia types 1 and 2: a proposed algorithm for limited resource-settings
Source: Front Neurol. 2026 Apr 21;17:1735246. doi: 10.3389/fneur.2026.1735246 (PMC13141855; doi:10.3389/fneur.2026.1735246)

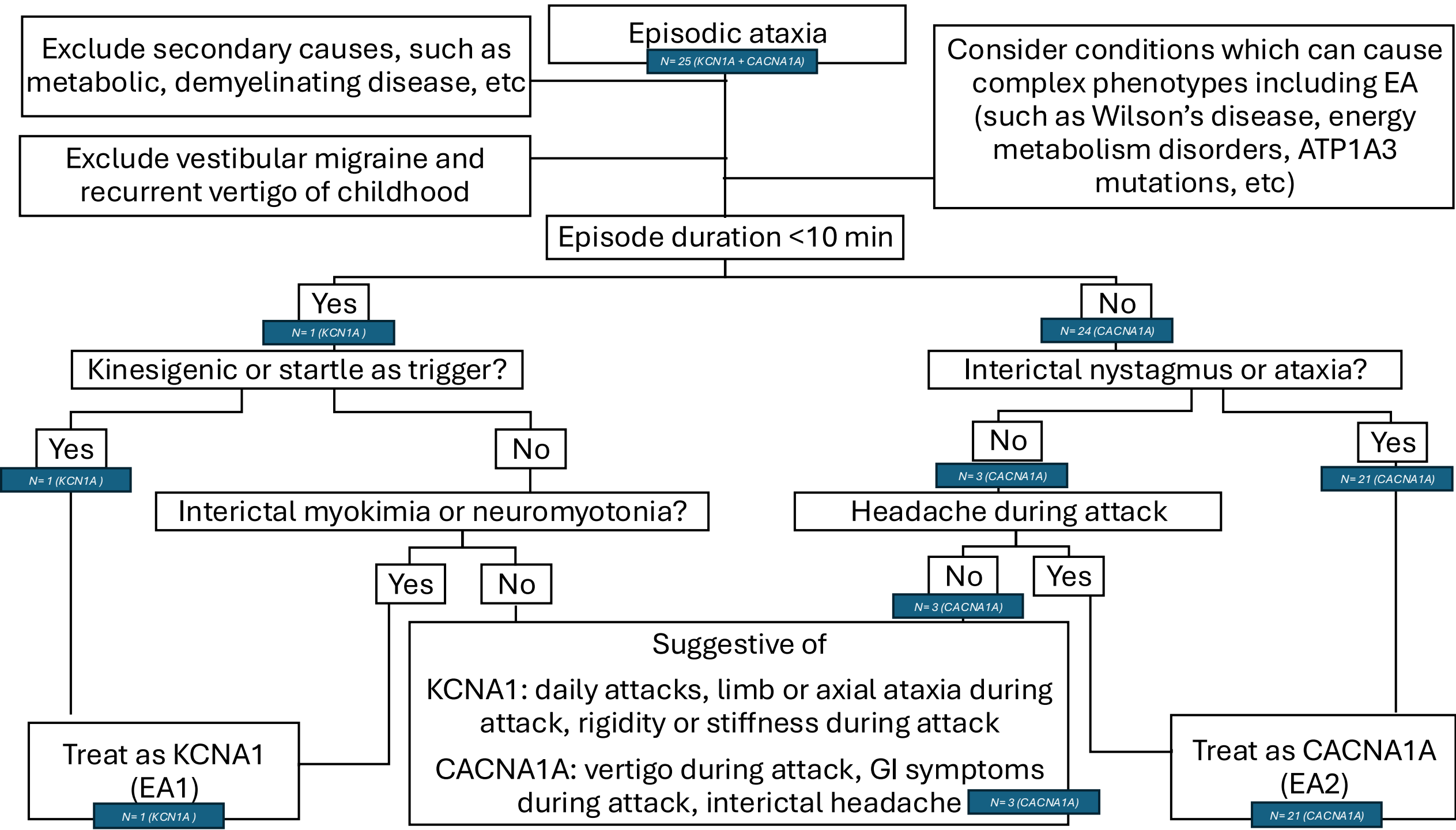

Supplement: Supplementary file 5 [file Supplementary_file_5.pdf]
